# Tailored charge-neutral self-assembled L_2_Zn_2_ container for taming oxalate

**DOI:** 10.3762/bjoc.20.250

**Published:** 2024-11-18

**Authors:** David Ocklenburg, David Van Craen

**Affiliations:** 1 Department of Chemistry and Chemical Biology, TU Dortmund University, Otto-Hahn-Straße 6, 44227 Dortmund, Germany

**Keywords:** anion recognition, charge-neutral host, metallocontainer, oxalate

## Abstract

Dicarboxylic acids and their derivatives play crucial roles in various biological processes, necessitating the development of effective receptors for their detection. In particular, the smallest dicarboxylate, oxalate, presents a significant importance due to its widespread presence in nature and its association with various diseases. Yet, very little attention was devoted to the recognition of oxalate with metal-driven self-assemblies like cages or containers while numerous classic organic receptors for oxalate exist. This discrepancy is astonishing because metallocontainers or metallocages have advantages over classic macrocycles or organocages like a higher modularity and good preorganization paired with a ready receptor preparation by metal complexation. The reason for the underrepresentation is the competitive nature and excellent ligand properties of oxalate which not only is associated with the aforementioned diseases but also poses a serious hazard for metal-driven self-assemblies because the dianion can easily replace ligand strands leading to a partial or full receptor decomposition. Herein, we present a charge-neutral zinc(II)-based metallocontainer which was tuned to contest oxalate as most competitive dicarboxylate. The dianion is bound in a 1:1 fashion with a binding constant of log *K* = 4.39 selectively over other dicarboxylates by maintaining the receptor stability. This study highlights the importance of a highly modular receptor design so that tailored hosts can be designed to tackle the recognition of challenging competitive analytes.

## Introduction

Dicarboxylic acids and their corresponding anions are essential intermediates in the biosynthesis of proteins and important biological metabolites [1–2]. As a result, the development of receptors for this class of compounds is of high interest [3–5]. Oxalate, the conjugated base of the smallest dicarboxylic acid, stands out from the list of highly relevant analytes because oxalic acid is found in common edible plants and its corresponding dianion is tied to several diseases, e.g., the formation of kidney stones through complexation of calcium and precipitation of calcium oxalate [6–8]. Unsurprisingly, the detection of oxalate was studied with numerous classic organic receptors involving acyclic hosts [9–21], macrocycles [22–33], and cages [34–44]. Additionally, organic receptors featuring metal centers which are strongly bound in a multidentate fashion with the sole purpose of acting as binding sites are utilized beside the pure organic systems [26,45–55].

In the last decade, metallocages and metallocontainers which are formed by metal-driven self-assembly have become especially popular to bind various kinds of anions since such systems offer easy to tune confinements. Usually, the utilized complexes are net positive which makes them ideal hosts for anions [56–63]. However, the inevitable presence of counteranions necessary to balance the positive charge increases the complexity of the underlying equilibria. Competition between the desired guest and the counteranions can lead to significantly weakened binding strength of the target analyte in the worst case. One option to mitigate this issue is to exchange the counteranions with less competitive ones, such as replacing tetrafluoroborate with tetrakis[3,5-bis(trifluoromethyl)phenyl]borate [64]. Alternatively, an emerging solution is the utilization of ligand–metal combinations that self-assemble into charge-neutral host systems free of accompanying counterions or other byproducts. Our group is pushing the boundaries of this approach, with each design starting with two important considerations. First, the counteranion of the metal precursor should be able to deprotonate the ligand, and the resulting corresponding acid needs to be volatile to simplify complex purification. Second, the solubility of the neutral host complexes usually needs to be improved by the installation of solubility groups, which must be considered during ligand design.

**Figure 1:**
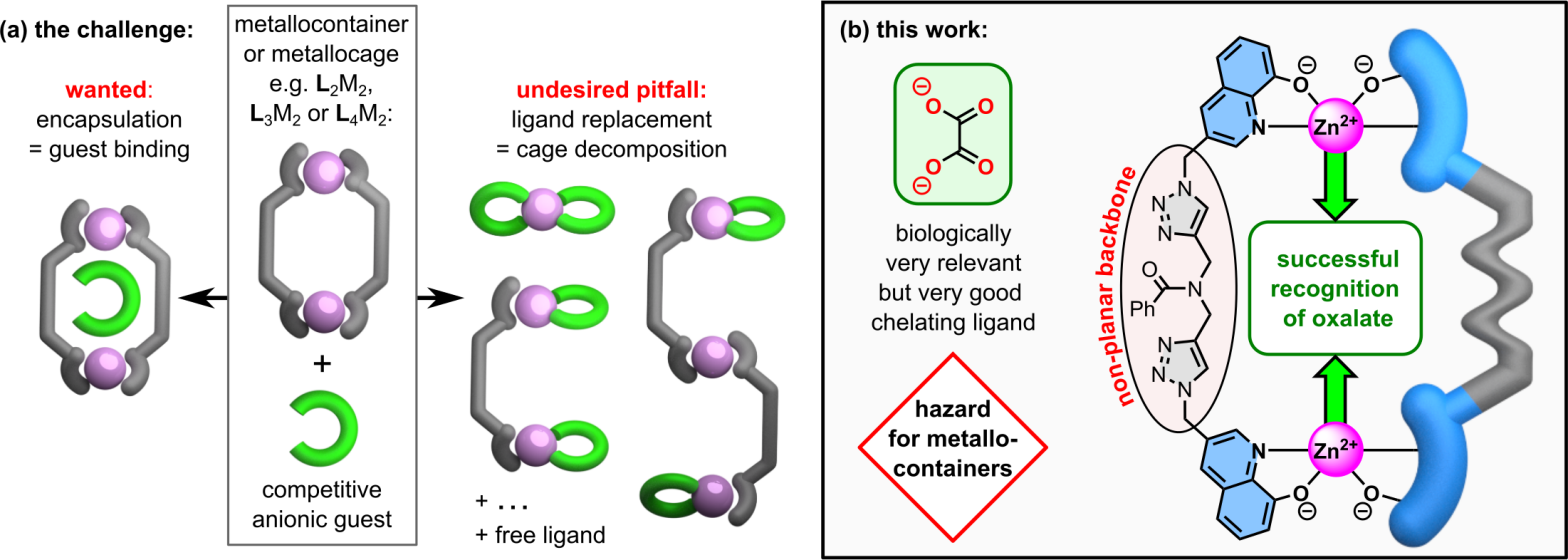
a) Generally desired guest recognition by encapsulation of the analyte within self-assembled metal-based containers or cages (left reaction). Often observed challenge with very competitive anionic guests which tend to result in decomposition of the initial receptor structure (right reaction). b) Performed backbone modification to tackle this issue and to tame oxalate as the most competitive dicarboxylate guest with a charge-neutral metallocontainer.

The biggest strength of metallocages and metallocontainers, whether they are positively charged or charge-neutral, is their metal-driven self-assembly. Unfortunately, this can also be their Achilles' heel, as the underlying coordination bonds are fragile compared to covalent bonds. For example, metallocages can easily break apart by the addition of competitive anions which are highly coordinating or which can act as good chelating ligands [65]. The latter holds true for oxalate as shortest dicarboxylate anion which likely forms complexes with all sorts of metals [66–67]. In fact, oxalate’s ideal ligand character is one reason for the aforementioned diseases and this property poses a fatal hazard for metallocages and metallocontainers. The dianion is capable of competing with the ligands used for self-assembled metal-based receptors instead of being bound inside the host structure as intended. Oxalate can strip off metal centers from the designed receptor causing a full or partial disassembly of the host structure. Guest-driven decomplexation events can also be utilized as detection method [65,68–73] but such processes are generally not desired for containers or cages because guest binding is anticipated to take place by encapsulation while the structure of the assembly is maintained. The undesired outcome is free ligand strands accompanied by simple metal oxalate complexes or unidentifiable mixtures of aggregates (Figure 1a). We observed this issue by ourselves for a self-assembled charge-neutral zinc-based container which proved otherwise to be an excellent dicarboxylate receptor by binding these dianions in a coordinative fashion between the zinc centers with binding constants up to log *K* = 8.16 [74]. However, even this very potent host was struggling with shorter dicarboxylates and with oxalate especially, resulting in an unknown mixture of species. In general, only a few reports with the focus on host–guest binding studies exist in which classic cationic metal-driven self-assemblies seem to be capable of binding oxalate without suffering from its competitive nature [75–79]. Guest encapsulation was investigated with cationic metalla-rectangles as well as metalla-bowls [76–79] resulting in binding constants of log *K* = 3.60 [76], 4.60 [77], and 3.48 [78] for oxalate binding in methanol. Additionally, a crystal structure of a positively charged copper(II) helicate with encapsulated oxalate was reported in a study with the focus on catalysis [80].

Inspired by these reports, our goal was to overcome the competitive nature of oxalate by encapsulating it in a charge-neutral metallocontainer for the first time. We revisited our previous receptor design and it was clear that the zinc–zinc distance needs to match the size of oxalate in order to tightly lock the dianion between the metal centers. We envisioned that a powerful lever for achieving a shorter metal–metal distance in our metallocontainer is to enhance the degree of freedom of the ligand backbone’s central part while maintaining the proven design principles with triazole connecting units for an easy ligand preparation (Figure 1b). The resulting compressed self-assembled host with a non-planar ligand backbone is the first charge-neutral metallocontainer able to bind oxalate forming a 1:1 host–guest complex with a binding constant of log *K* = 4.39 ± 0.04 in DMSO as highly competitive solvent. Thus, the tailored receptor can compete with the closely related literature examples in terms of oxalate-binding strength even though much weaker ion–ion attraction is present compared to positively charged metallacycles or cages which bind anionic guests.

## Results and Discussion

**Scheme 1:**
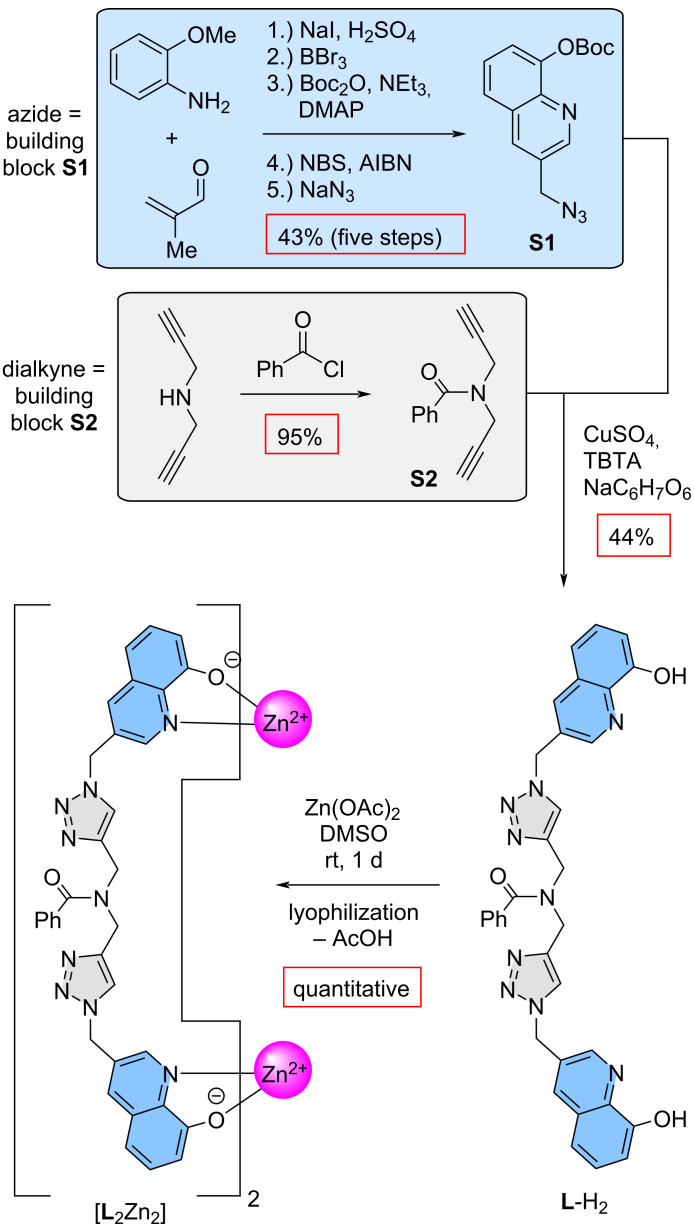
Two-synthon approach for ligand preparation via CuAAC click reaction of an azide-functionalized, protected 8-hydroxyquinoline and a dialkyne based on dipropargylamine. The double-stranded [**L**_2_Zn_2_] receptor is obtained via self-assembly of two ligand strands with zinc(II) acetate followed by lyophilization.

**Ligand preparation and host assembly.** The synthesis adheres to a two-synthon approach which relies on the connection of two building blocks through a CuAAC click reaction (Scheme 1). Building block **S1**, the azide, is not modified in this work and the preparation is achieved with an overall yield of 43% via the previously reported methods. Building block **S2**, based on dipropargylamine, is synthesized via a Schotten–Baumann reaction with benzoyl chloride, resulting in the flexible dialkyne backbone with a yield of 95%. The functionalization was envisioned to enhance the solubility of the ligand as we usually have troubles with the formed complexes in this respect. The final step of the ligand preparation is the CuAAC click reaction. The protecting Boc group of the 8-hydroxyquinoline units was cleaved during the workup right away resulting in ligand **L**-H_2_ with 44% yield. Self-assembly in DMSO with zinc(II) via its acetate salt results in a charge-neutral and bench-stable [**L**_2_Zn_2_] metallocontainer. Purification of the complex is achieved by lyophilization which accomplishes the removal of acetic acid as only byproduct of the complexation reaction and DMSO as solvent. The charge-neutral receptor is obtained as a yellow solid from this process in quantitative yield and the complex is soluble in DMSO as intended.

The signal of the methylene group (H_f_) adjacent to the quinolate moiety shows splitting upon complex formation (Figures S3 and S4 in Supporting Information File 1). This observation is a known indication for the formation of a racemic helicate which usually can result in diastereotopic splitting of methylene groups which are in the direct periphery of the metal complex units. Furthermore, the triazole signals (H_g_) of the [**L**_2_Zn_2_] complex are also split. A possible cause for this rather unexpected behavior is a rotational barrier of the phenylamide residue which is visible for the complex [**L**_2_Zn_2_] and the free ligand **L**-H_2_ due to splitting of the neighboring methylene unit (H_h_).

An important sidenote is that preliminary tests with the deprotected click product of non-functionalized dipropargylamine as ligand resulted in no clean formation of a charge-neutral metallocontainer with zinc(II) acetate. We believe that the modification with benzoyl chloride does not only alter the solubility but also shields the secondary amine which might act as additional coordination site.

**Binding of monocarboxylate anions and oxalate.** Acetate and benzoate as monocarboxylates were investigated alongside oxalate because we expected also a different binding scenario for these small guests as a result of the altered zinc–zinc distance compared to our previous study [74]. Indeed, ^1^H NMR host–guest titrations showed a different behavior for acetate and benzoate which form only 1:1 complexes with the new [**L**_2_Zn_2_] receptor (Figures S6–S17 in Supporting Information File 1) in contrast to the observed 1:2 binding fashion seen before [74]. This twist is a promising hint towards our goal of binding oxalate inside the [**L**_2_Zn_2_] receptor because the modification seems to decrease the zinc–zinc distance and the cavity size substantially enough to prevent coordination of monocarboxylates to both zinc centers due to their sterical demand. Average binding constants of log *K* = 3.44 for acetate and log *K* = 3.33 for benzoate were determined by NMR (see Supporting Information File 1, Figures S7, S9, S11, S13, S15, and S17).

**Figure 2:**
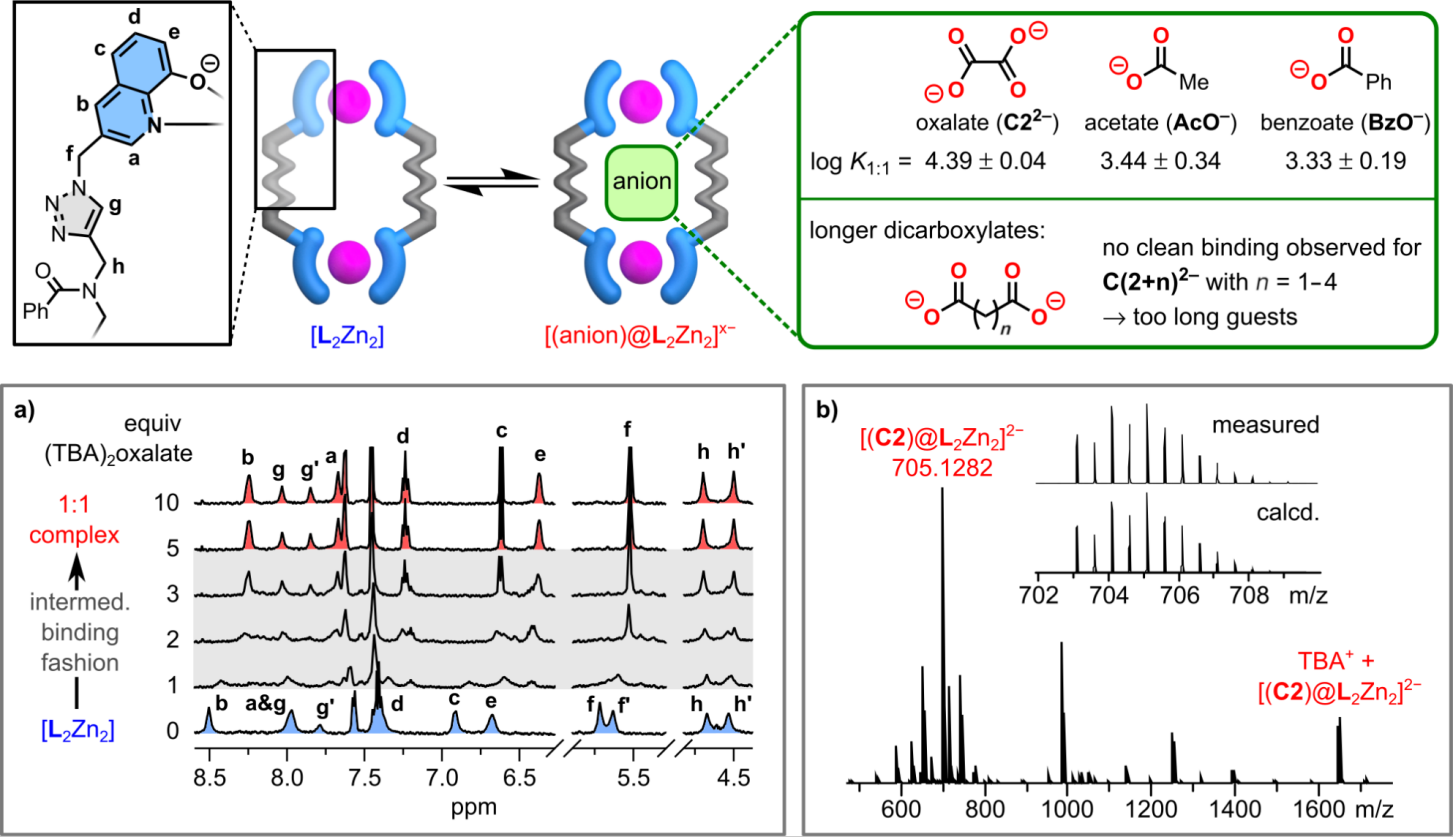
Recognition of mono- and dicarboxylates with [**L**_2_Zn_2_] which results in the formation of 1:1 host–guest complexes in case of clean binding which is observed for acetate, benzoate, and oxalate. a) ^1^H NMR titration (500 MHz, 500 µM, DMSO-*d*_6_, 25 °C) of [**L**_2_Zn_2_] with oxalate showing intermediate-like exchange. b) Negative ESI-MS spectrum of [**L**_2_Zn_2_] with 5 equiv oxalate showing the formation of [(**C2**)@**L**_2_Zn_2_]^2−^ as host–guest complex.

^1^H NMR dicarboxylate binding studies were carried out with oxalate (**C2^2−^**) and longer variants **C(2+*n*)^2−^** up to adipate (with *n* = 4). Oxalate addition to [**L**_2_Zn_2_] results in an intermediate exchange with broadened NMR signals at the beginning of the titration. The signals become sharp again after 3 equiv of oxalate were added (Figure 2a). The observation is a full success because of two reasons. On the one hand, the well-defined signals are indicating a defined host–guest complex species, ruling out the formation of undesired unknown aggregates. On the other hand, the ^1^H NMR spectrum does not match the one of the free ligand which shows that neither full metal extrusion took place as a result of the competitive nature of oxalate.

**Figure 3:**
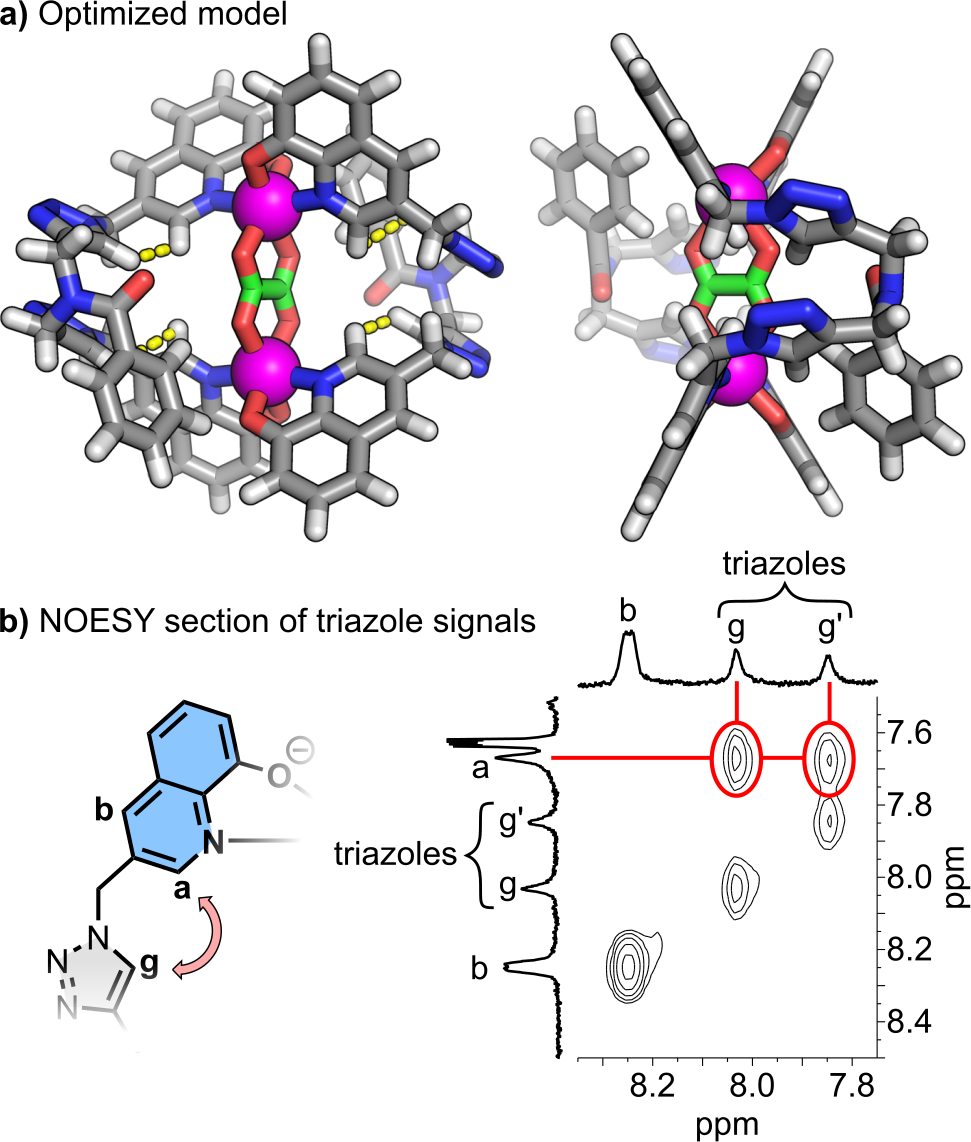
a) Optimized structure (ωB97X‒D4 [83]/def2-SVP [84–85], implicit solvation with DMSO [86], ORCA 5.0.3 [81–82]) of [(**C2**)@**L**_2_Zn_2_]^2−^ showing a “*meso*-helicate” structure with oxalate coordinating to both zinc centers in a chelating fashion. b) 2D NOE contacts between H_triazole_ and H_quinolate_ (H_g_···H_a_).

Negative ESI-MS of a host–guest mixture with 5 equiv of oxalate gave the final proof that a distinct 1:1 host–guest complex was formed (Figure 2b). The major signal at *m*/*z* of 705.1282 and its isotope pattern matches with the calculated value (705.1263) of the 1:1 complex [(**C2**)@**L**_2_Zn_2_]^2‒^. The *m*/*z*-signal at 1652.5394 fits to a single negatively charged TBA adduct of the 1:1 complex (Figure S20 in Supporting Information File 1). An average binding constant for oxalate of log *K* = 4.39 was obtained by UV–vis spectroscopy (Figures S21–S26 in Supporting Information File 1) since the intermediate-like exchange prevents the determination directly from the ^1^H NMR titration. The binding constant of oxalate is an order of magnitude higher than the one of acetate and benzoate which is in line with the fact that oxalate is supposed to be pinched between both zinc centers while the monocarboxylates coordinate only to one zinc atom. Unfortunately, we could not grow suitable single crystals of sufficient quality to obtain a solid-state structure of the oxalate host–guest complex [(**C2**)@**L**_2_Zn_2_]^2−^ so that we utilized DFT calculations to model the expected structure of the host–guest species (Figure 3a). Therefore, we funneled structural information obtained from NMR spectra into our starting geometry. First, the signals of the peripheral methylene group (H_f_) of the quinolinate units show diastereotopic splitting for empty [**L**_2_Zn_2_] which points towards the existence of a racemic (ΔΔ and ΛΛ) helicate. These signals merge upon oxalate binding (Figure 2a) which is an indication that the 1:1 complex [(**C2**)@**L**_2_Zn_2_]^2−^ exists probably as *meso*-form and a helicate to “*meso*-helicate” transformation took place. Second, a 2D NOESY NMR spectrum of the 1:1 complex with oxalate reveals through-space contacts of the triazole protons (H_g_) with the aromatic proton H_a_ which is next to the coordinating nitrogen center of the quinolate group (Figure 3b). This information is evidence for the triazole units pointing with their proton towards the cavity. The geometry of the model was optimized (ORCA 5.0.3 [81–82]) using ωB97X‒D4 [83]/def2-SVP [84–85] with implicit solvent (DMSO) [86]. The triazole groups of each backbone adopt a nearly parallel orientation with a slight tilt compared to the horizontal axis which results in one short (Figure 3a, left ligand: 2.2 Å, and right ligand: 2.3 Å) and one longer H_triazole_···H_quinolate_ (H_g_···H_a_) distance (left ligand: 2.8 Å, and right ligand: 2.8 Å). Contradictory, the calculated distance from the NOE cross peaks is roughly 2 Å for both contacts.

We know that the triazole units can rotate by some degree at ambient temperature based on previous investigations [87]. Thus, dynamic tilting up and downwards of the triazole groups is expected. Molecular dynamics simulations (xTB 6.6.0 [88–89]) support this hypothesis (Figure S32 in Supporting Information File 1) and average H_triazole_···H_quinolate_ (H_g_···H_a_) distances of 2.6 Å, 2.3 Å, 2.3 Å, and 2.4 Å are found for the four through-space interactions over a timescale of 800 ps.

**Investigation of longer aliphatic dicarboxylate guests and competition experiments. **^1^H NMR titrations with malonate, succinate, glutarate, and adipate showed no clean binding (formation of mixtures) upon addition of these guests as TBA salt to [**L**_2_Zn_2_], with broad regions and multiple sets of signals persisting even after the addition of a large excess of the analytes (Table 1, and Figures S27–S30 in Supporting Information File 1). Binding constants for these dicarboxylates were not determined due to the lack of clarity regarding the underlying binding equilibria and the exact speciation. It seems like the receptor does not provide a suitable size match for these dicarboxylates in terms of the zinc–zinc distance and a selective formation of 1:1 host–guest species does not occur. Nitrate, sulfate, and dithionite showed no significant binding as examples of guests without carboxylate residues (Figure S31 in Supporting Information File 1).

**Table 1:** Overview of studied anionic guests as TBA salts and determined binding constants for host–guest recognition in DMSO-*d*_6_.

carboxylate anion	log *K*_1:1_

acetate (**AcO^−^**)	3.44 ± 0.34^a,b^
benzoate (**BzO^−^**)	3.33 ± 0.19^a,b^
oxalate (**C2^2−^**)	4.39 ± 0.04^b,c^
malonate (**C3^2−^**)	n.d.^d^
succinate (**C4^2−^**)	n.d.^d^
glutarate (**C5^2−^**)	n.d.^d^
adipate (**C6^2−^**)	n.d.^d^

^a^Average binding constant determined by three ^1^H NMR titrations (data fitted with BindFit [90–92]). ^b^Uncertainty represents the standard deviation of the multiple measurements. ^c^Average binding constant obtained by three UV–vis titrations (data fitted with HypSpec2014 [93]). ^d^Binding constant could not be determined because multiple unknown species are formed.

**Figure 4:**
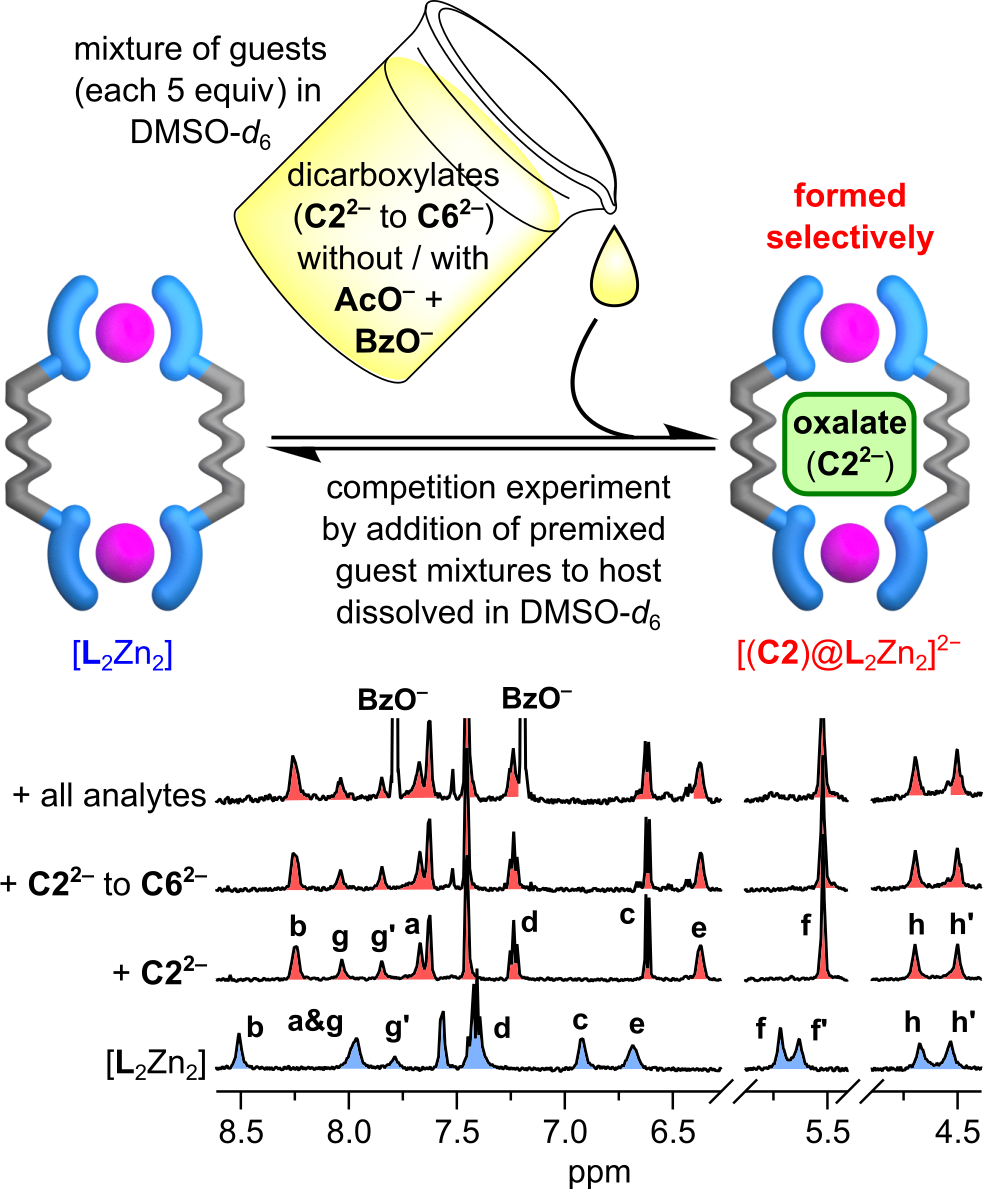
^1^H NMR competition experiments (500 MHz, 500 µM, DMSO-*d*_6_, 25 °C) with mixtures of studied analytes showing the selective formation of the 1:1 oxalate host–guest complex [(**C2**)@**L**_2_Zn_2_]^2−^ in all cases.

A qualitative comparison of the guest binding tendency was accomplished by ^1^H NMR competition experiments to find out about the selectivity of the new receptor and to circumvent the drawback that no binding constants for longer dicarboxylates could be determined. Therefore, the behavior of [**L**_2_Zn_2_] with different combinations of analytes added as mixtures with 5 equiv of each TBA salt was investigated (Figure 4). The recorded spectra after addition of all dicarboxylates (**C2^2^**^−^ to **C6^2−^**) with and without monocarboxylates (**AcO^−^** and **BzO^−^**) match the spectrum of [(**C2**)@**L**_2_Zn_2_]^2‒^ which was formed by addition of 5 equiv oxalate alone. The competition experiments clearly show that the host favors and binds oxalate selectively over all other guests of this study.

## Conclusion

Oxalate as highly competitive dianion with excellent coordinating and chelating properties was successfully recognized by a charge-neutral self-assembled zinc(II) metallocontainer through formation of a 1:1 host–guest complex with the [**L**_2_Zn_2_] receptor staying intact upon oxalate addition. This observation is not taken for granted for, e.g., cage-like metal-based self-assembled receptor systems which tend to become destabilized through highly competitive anions. Our approach showcases that an adjustment of, e.g., the cavity size is a powerful lever to tame guests which are hazardous for metal-based cages or containers so that an undesired full or partial receptor disassembly is ruled out. Binding of oxalate occurs with a binding constant of log *K* = 4.39 with a high selectivity over the other mono- and dicarboxylate guests of this study which was shown qualitatively by competition experiments. For the future, we aim at developing a universal charge-neutral zinc(II) container capable of binding oxalate as well as longer dicarboxylates in a clean fashion with an included lever to control the selectivity by an external stimulus. Additionally, the clear take home messages from recent conferences paired with the latest insights from working with such charge-neutral hosts in the laboratory show that one major task is to find a solution for the so far limiting solubility.

## Supporting Information

File 1 Synthetic protocols, characterization data, NMR spectra, guest binding investigations, data fitting, and computational modeling details.

## Data Availability

Data generated and analyzed during this study is available from the corresponding author upon reasonable request.
