# Supplementary material for: Tailored charge-neutral self-assembled L2Zn2 container for taming oxalate
Source: Beilstein J Org Chem. 2024 Nov 18;20:3007–15. doi: 10.3762/bjoc.20.250 (PMC11590019; doi:10.3762/bjoc.20.250)
Supplement: File 1 — Synthetic protocols, characterization data, NMR spectra, guest binding investigations, data fitting, and computational modeling details. [file Beilstein_J_Org_Chem-20-3007-s001.pdf]

BEILSTEIN JOURNAL OF ORGANIC CHEMISTRY

Supporting Information

for

Tailored charge-neutral self-assembled L_2Zn_2 container for taming oxalate

David Ocklenburg and David Van Craen

Beilstein J. Org. Chem. **2024,** *20,* 3007–3015. doi:10.3762/bjoc.20.250

Synthetic protocols, characterization data, NMR spectra, guest binding investigations, data fitting, and computational modeling details

License and Terms: This is a supporting information file under the terms of the Creative Commons Attribution License (https://creativecommons.org/licenses/by/4.0). Please note that the reuse, redistribution and reproduction in particular requires that the author(s) and source are credited and that individual graphics may be subject to special legal provisions.
The license is subject to the *Beilstein Journal of Organic Chemistry* terms and conditions: (https://www.beilstein-journals.org/bjoc/terms)

1. Materials and methods

Solvents and chemicals were obtained from Alfa Aesar, abcr, TCI, chemPUR and VWR. $CDCl_3$ and DMSO-d_6 from Eurisotop were used for binding studies and characterization of products. Column chromatography was performed with silica gel $60\,Å$ (0.06–$0.2\,mm$). NMR spectroscopy (^{1}H, ^{13}C, COSY, NOESY, HSQC, HMBC and VT) was performed with a $500\,MHz$ Bruker Avance NEO NMR spectrometer. High-resolution mass spectrometry was conducted using a Bruker timsTOF spectrometer and UV-Vis spectroscopy was accomplished using an Agilent HP-8453 UV-Vis (DAD) instrument.

2. Synthetic procedures

2.1. 3-(Azidomethyl)quinolin-8-yl *tert*-butyl carbonate (S1)

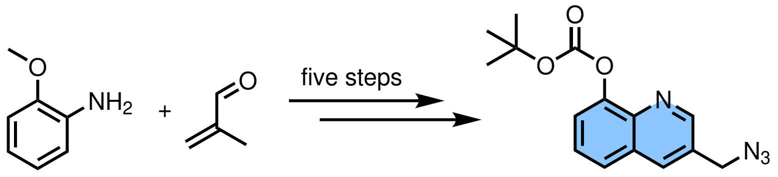

1 **2** **S1**

The azide **S1** was prepared in five steps according to the synthetic route starting from *o*-anisidine (**1**) and methacroleine (**2**) reported by Van Craen and co-workers.[1]

2.2. *N*,*N*-Di(prop-2-yn-1-yl)benzamide (S2)

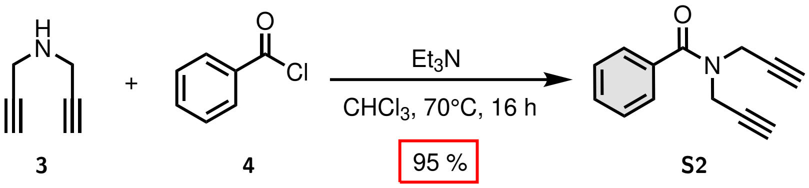

3 **4** **S2**

Dipropargylamine (**3**, $1.11\,mL$, $10.74\,mmol$, 3 equiv) was dissolved in chloroform ($50\,mL$) and triethylamine ($1.49\,mL$, $10.74\,mmol$, 3 equiv) was added. After 5 min of stirring benzoyl chloride (**4**, $0.41\,mL$, $3.58\,mmol$, 1 equiv) was added slowly and the reaction mixture was stirred over night at $70\,°C$. Then the reaction mixture was quenched with water. Afterwards the aqueous phase was extracted three times with chloroform. The combined organic phase was dried over magnesium sulfate and the solvent was evaporated under reduced pressure. Column chromatography (silica, pentane/ethyl acetate 9:1) gives *N*,*N*-di(prop-2-yn-1-yl)benzamide (**S2**) as a yellowish oil, which crystallizes in the fridge (95%, $668.9\,mg$, $3.39\,mmol$). The ^{1}H NMR data is in line with the reported values in the literature.[2,3,4]

^{1}H NMR ($500\,MHz$, $CDCl_3$, $25\,°C$): δ = 7.59-7.53 (m, 2H), 7.50-7.40 (m, 3H), 4.47 (br. s, 2H), 4.20 (br. s, 2H), 2.32 (br. s, 2H) ppm.

2.3. *N,N*-Bis((1-((8-hydroxyquinolin-3-yl)methyl)-1*H*-1,2,3-triazol-4-yl)methyl)benzamide (L-H_2)

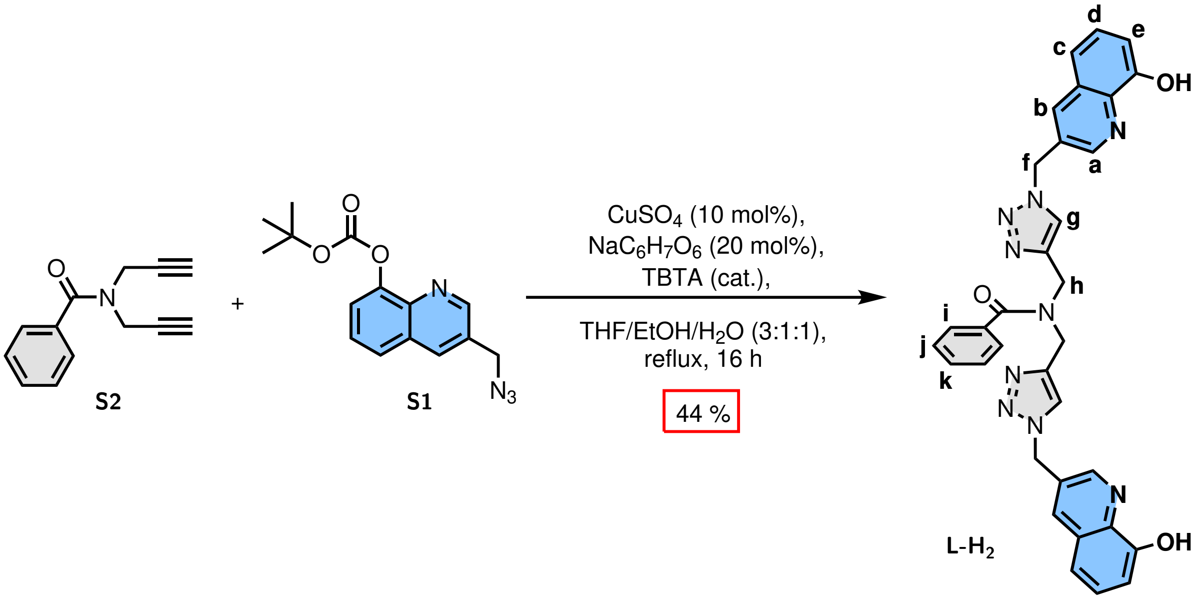

The azide (**S1**, 85.4 mg, 284 µmol, 2.2 equiv) was dissolved in a mixture of THF/EtOH/H_2O (3:1:1, 10 mL, 0.01 M) and transferred into a Schlenk tube. The solution was degassed for 20 min with argon. $CuSO_4$ (2 mg, 13 µmol, 0.1 equiv), sodium ascorbate (12.8 mg, 64.6 µmol, 0.5 equiv) and TBTA (0.7 mg, 1.3 µmol, 0.01 equiv) were sequentially added. Benzamide (**S2**, 25.5 mg, 129 µmol, 1 equiv) was added under argon and the reaction solution was heated to 100 °C and stirred for 16 h in a closed tube. The reaction solution was diluted with chloroform and the organic phase was washed with EDTA (0.25 mM), brine and saturated ammonium chloride solution. The aqueous phase was extracted with chloroform. The combined organic phase was dried with $MgSO_4$ and the solvent was removed under reduced pressure. Column chromatography (silica, DCM/MeOH gradient 0 % - 5 %) yields the ligand **L**-H_2 as a brown-greenish solid (44 %, 34.3 mg, 57.4 µmol). The Boc-protection groups were cleaved during the workup right away.

^{1}**H NMR** (600 MHz, DMSO-d_6, 25 °C): δ = 9.92 (s, 2H, OH), 8.84 (s, 2H, H_a), 8.21 (s, 2H, H_g), 8.19 (s, 2H, H_b), 7.52 (d, J = 6.9 Hz, 2H, H_i), 7.46-7.37 (m, 7H, H_j, H_k, H_d and H_c), 7.09 (d, J = 7.5 Hz, 2H, H_e), 5.81 (s, 4H, H_f), 4.63 (s, 2H, H_h), 4.49 (s, 2H, $H_{h'}$) ppm. ^{13}**C NMR** (151 MHz, DMSO-d_6, 25 °C): δ = 170.50, 153.40, 148.07, 143.27, 137.96, 135.84, 135.10, 129.66, 129.48, 128.35, 128.23, 128.16, 126.88, 123.98, 117.77, 111.88, 50.51, 43.91 ppm. **HRMS** (positive ESI-MS, MeCN, acidified): m/z = 598.2339 (M + H^+, $C_{33}H_{28}N_9O_3^+$, calcd. 598.2310), 620.2112 (M + Na^+, $C_{33}H_{27}N_9O_3Na^+$, calcd. 620.2129).

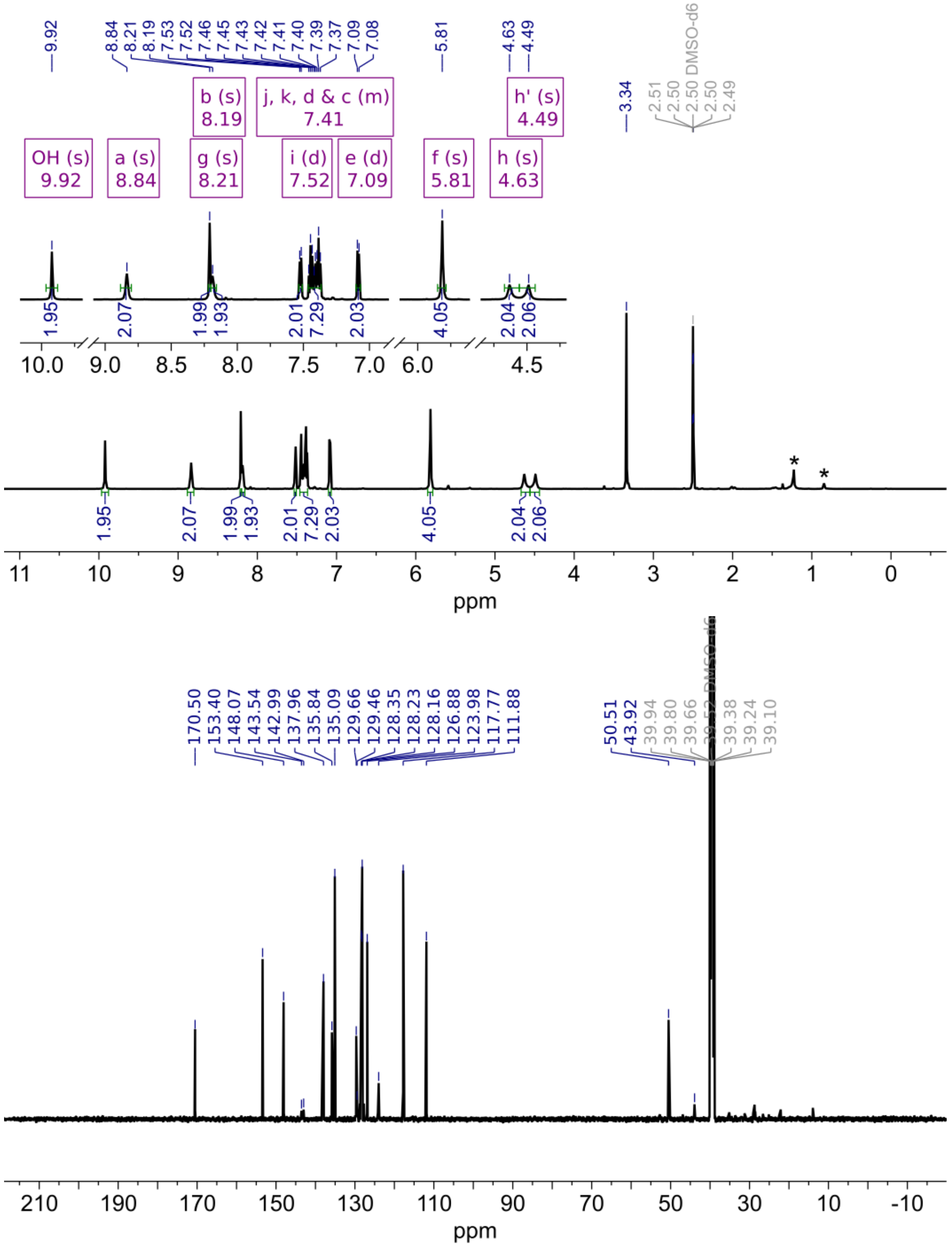

Figure S1: ^{1}H (top) and ^{13}C NMR spectrum (bottom) of ligand **L**-H_2. Signals labeled with a ∗ refer to grease. Splitting of C_h (one signal is overlapping with DMSO, see HSQC) and the quaternary triazole carbon atoms is observed in the ^{13}C NMR spectrum.

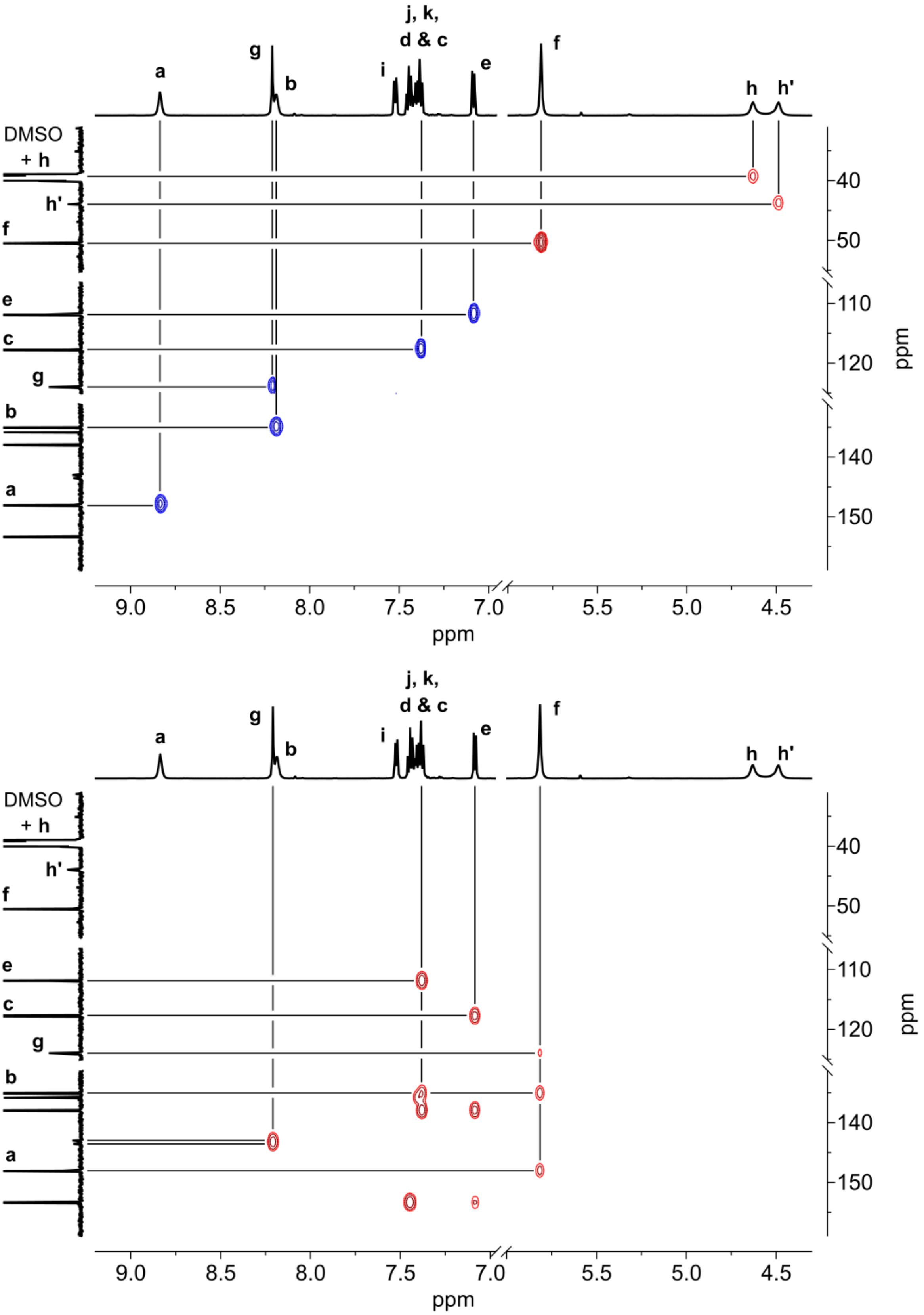

Figure S2: Relevant section of the HSQC (top) and HMBC NMR spectrum (bottom) of ligand **L**-H_2 which is used for signal assignement.

2.4. Self-assembly of the charge-neutral [L_2Zn_2] complex

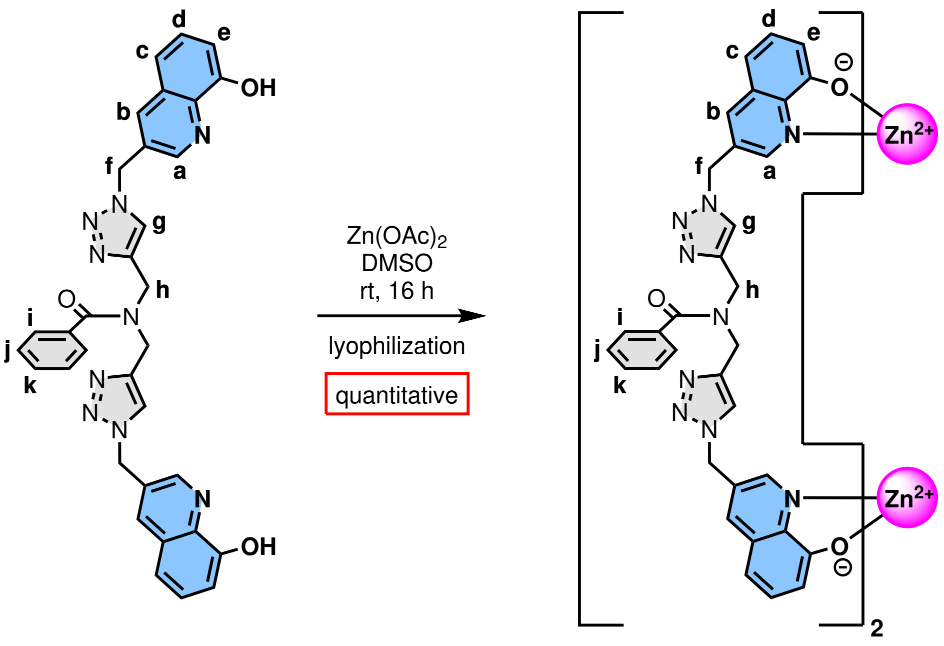

Ligand **L**-H_2 (1 equiv) and $Zn(OAc)_2$ (1 equiv) were dissolved in DMSO (500 μM). The zinc acetate solution was added to the stirring ligand solution which results in a colour change to yellow. The solution was allowed to stir for 16 h and the solvent as well as the byproduct acetic acid were removed via lyophilisation, which yields the complex as a yellow solid. The complex is suspended and washed with MeOH for further purification. The mixture is centrifuged and the solvent is removed by decantation. This cleaning process is repeated twice. The resulting precipitate was again dissolved in DMSO which was removed via lyophilization to yields the clean complex as a yellow solid in a quantitative fashion.

^{1}H NMR (500 MHz, DMSO-d_6, 25 °C): δ = 8.51 (s, 4H, H_b), 7.97 (br., 6H, H_a and H_g), 7.78 (s, 2H, $H_{g'}$), 7.57 (d, J = 7.2 Hz, 4H, H_i), 7.47-7.36 (m, 10H, H_d, H_j and H_k), 6.92 (d, J = 6.9 Hz, 4H, H_c), 6.68 (br., 4H, H_e), 5.72 (s, 4H, H_f), 5.63 (s, 4H, $H_{f'}$), 4.67 (s, 4H, H_h), 4.53 (s, 4H, $H_{h'}$) ppm. **HRMS** (negative ESI-MS, MeCN/DMSO): m/z = 1357.2493 ([M + Cl^-], [(**Cl**)@**L**$_2$Zn$_2$]$^-$, [(Cl)@($C_{33}H_{25}N_9O_3$)$_2$Zn$_2$]$^-$, calcd. 1357.2408).

Variable temperature ^{1}H NMR studies (Figure S4) and NOESY as well as COSY measurements (Figure S5) at high temperature were conducted because the signal assignement is hampered at room temperature as a result of the in general broad and partially overlapping signals.

^{1}H NMR (500 MHz, DMSO-d_6, 90 °C): δ = 8.47 (s, 4H, H_b), 8.09 (s, 4H, H_a), 7.78 (s, 4H, H_g), 7.51 (dd, J = 8.0, 1.4 Hz, 4H, H_i), 7.44-7.36 (m, 10H, H_d, H_j and H_k), 6.93 (d, 7.9 Hz, 4H, H_c), 6.76 (d, 7.8 Hz, 4H, H_e), 5.66 (s, 8H, H_f), 4.63 (s, 8H, H_h) ppm.

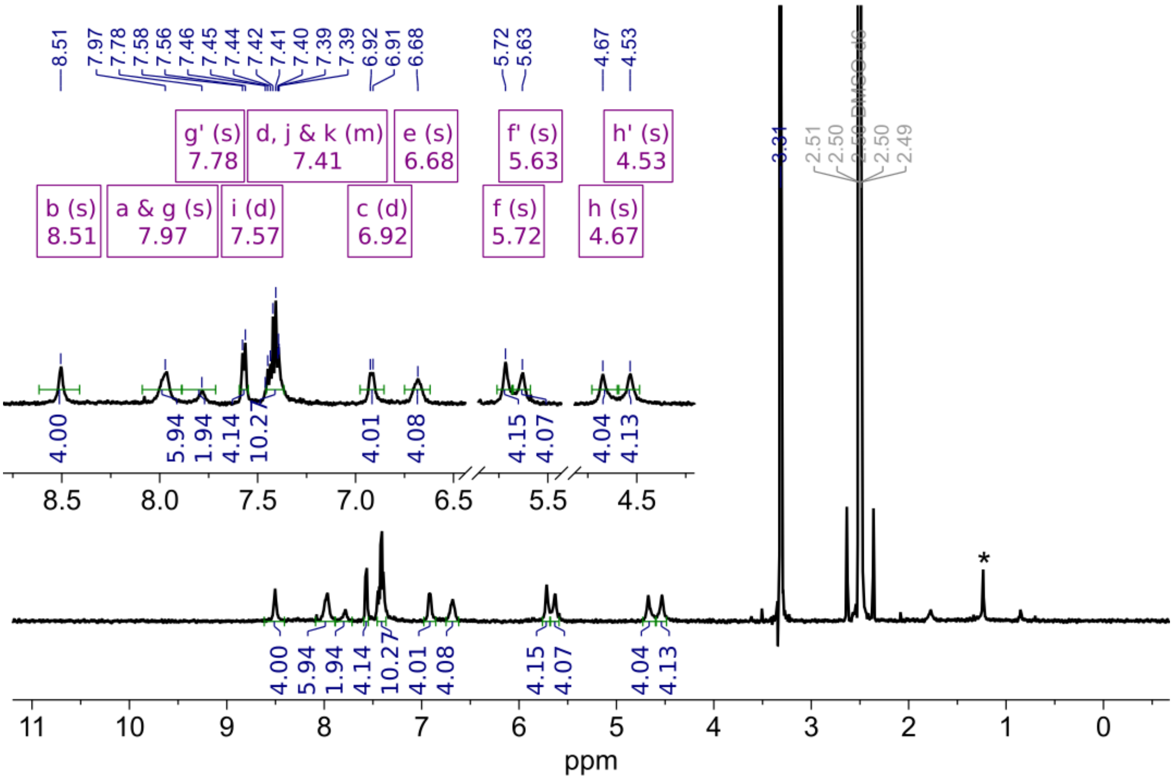

Figure S3: ^{1}H NMR of [$\mathbf{L}_2Zn_2$] in DMSO-d_6 at $25\,°C$. The signal labeled with a $*$ is grease.

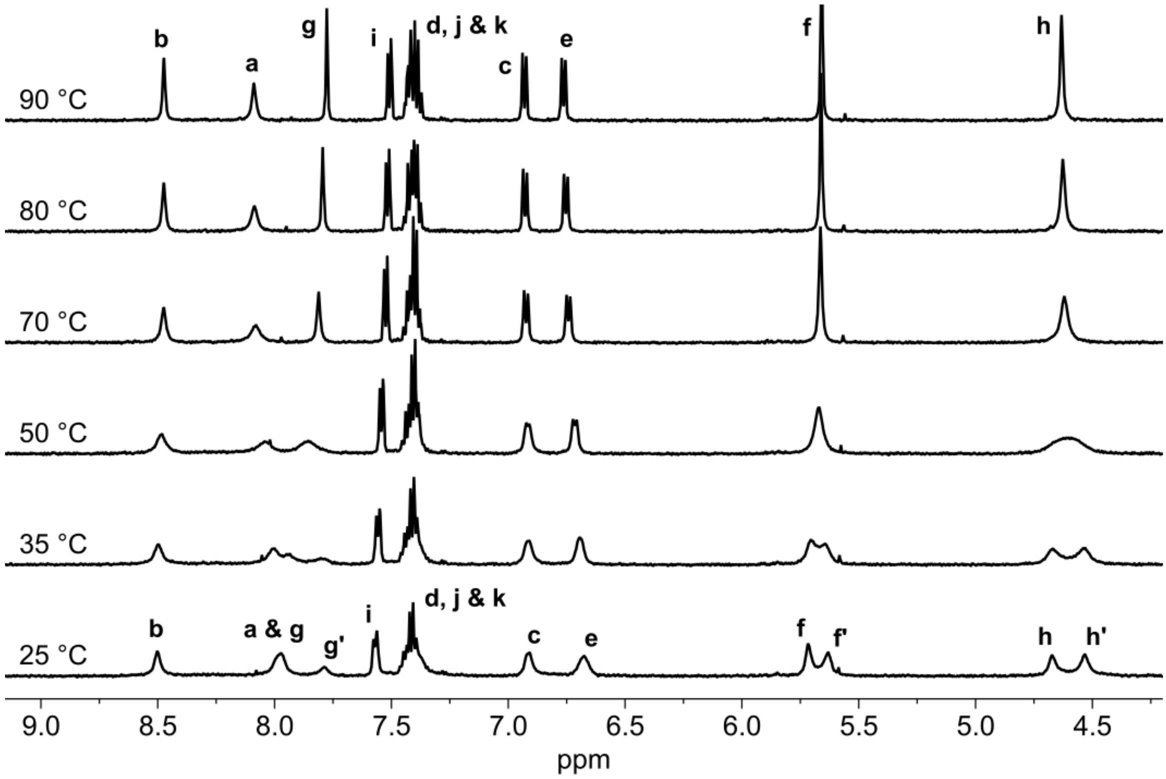

Figure S4: ^{1}H NMR of [$\mathbf{L}_2Zn_2$] in DMSO-d_6 at different temperatures ranging from $25\,°C$ to $90\,°C$.

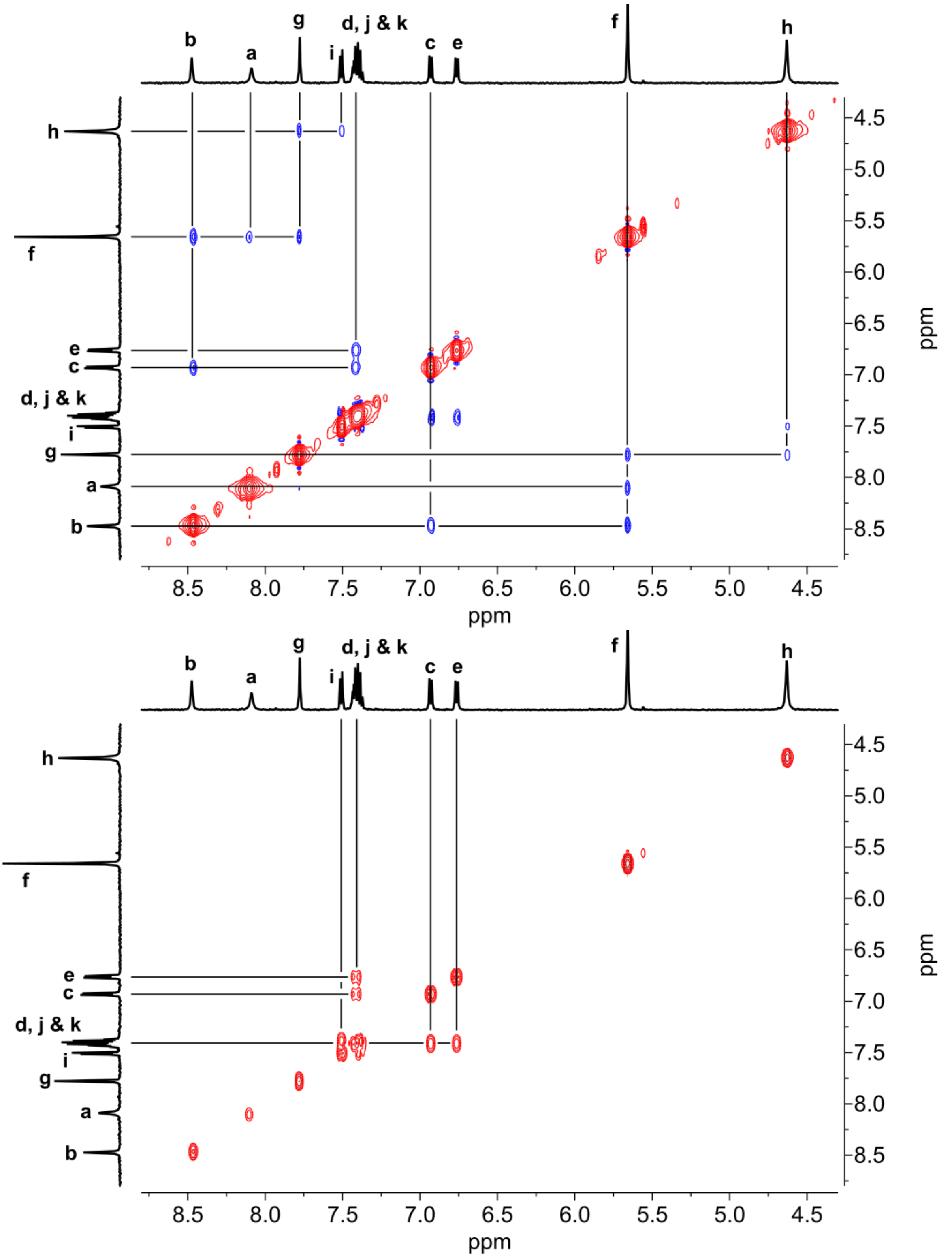

Figure S5: NOESY (top) and COSY (bottom) NMR spectrum of [$\mathbf{L}_2Zn_2$] in DMSO-d_6 at $90\,^{\circ}\mathrm{C}$.

3. Anion binding studies

3.1. Procedures

Deprotonation of dicarboxylic acids

Oxalic, malonic, succinic, glutaric and adipic acid were commercially obtained from chemPUR and Merck (Sigma–Aldrich). The dicarboxylic acids were dissolved in a small amount of methanol (up to $5\,\mathrm{mL}$) and two equivalents of tetrabutylammonium hydroxid ($1\,\mathrm{M}$ in MeOH) was added to deprotonate the acids. Afterwards, the mixture was vortexed for $30\,\mathrm{min}$. MeOH was removed under reduced pressure after the mixing time. A small amount of water (1–$2\,\mathrm{mL}$) was added to the remaining slurry to assist the final lyophilizations process since the available device is not rated for pure methanol (too low melting point). The tetrabutylammonium (TBA) dicarboxylates were used without further workup after several days of lyophilization.

Titration procedure

All binding studies were performed dilution corrected. Binding constants for monocarboxylates (acetate and benzoate) were determined by ^{1}H NMR titrations since a fast-exchange behavior is found for these guests. The obtained data was fitted by BindFit.[5,6,7] The binding constant for oxalate was obtained by performing UV–vis titrations (Hellma 10 mm x 10 mm QS cuvettes, room temperature) because of the intermediate-like exchange behavior in the NMR. Therefore, the absorbance between $290\,\mathrm{nm}$ and $550\,\mathrm{nm}$ was fitted globally with the program HypSpec2014.[8] Errors were estimated by repeating the NMR and UV–vis titration experiments three times.

Overview of determined binding constants

Table S1: Binding data for [$\mathbf{L}_2$Zn$_2$] with acetate, benzoate and oxalate.

guest (TBA salt)	iteration	log $K_{1:1}$	average log $K_{1:1}$	%error (standard deviation)
	1	3.41		
acetate (**AcO$^-$**)^a	2	3.80	3.44	± 0.34
	3	3.12		
	1	3.50		
benzoate (**BzO$^-$**)^a	2	3.36	3.33	± 0.19
	3	3.13		
	1	4.36		
oxalate (**C2^{2-}**)^b	2	4.44	4.39	± 0.04
	3	4.37		

Binding constants determined by (a) ^{1}H NMR titrations with BindFit[5,6,7] and (b) UV–vis titrations with HypSpec2014.[8]

3.2. Acetate

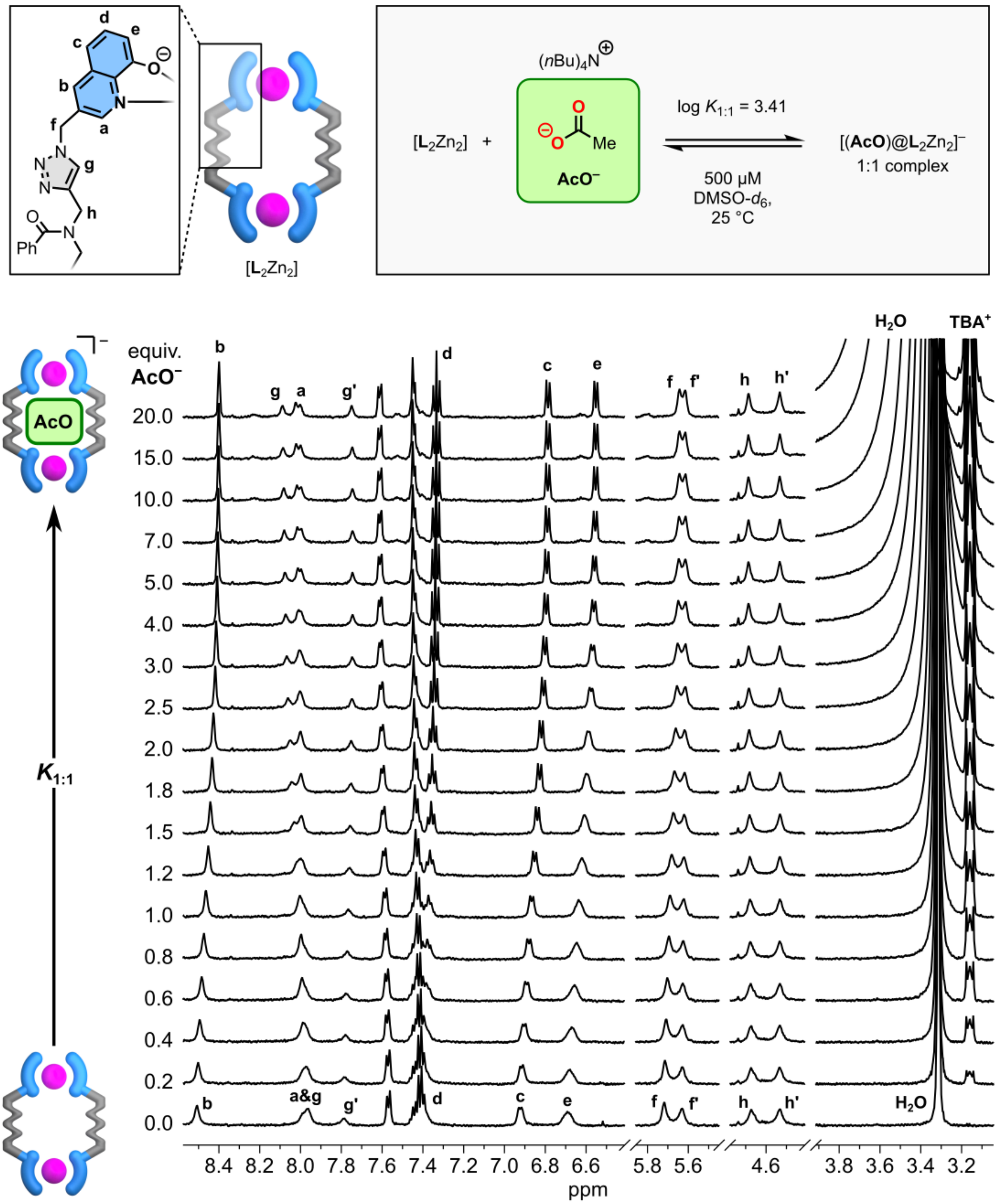

Figure S6: ^{1}H NMR titration ($500\,\mathrm{MHz}$, $500\,\mu\mathrm{M}$, DMSO-d_6, $25\,^\circ\mathrm{C}$) of [$\mathbf{L}_2$Zn$_2$] with tetrabutylammonium acetate (**AcO**$^-$).

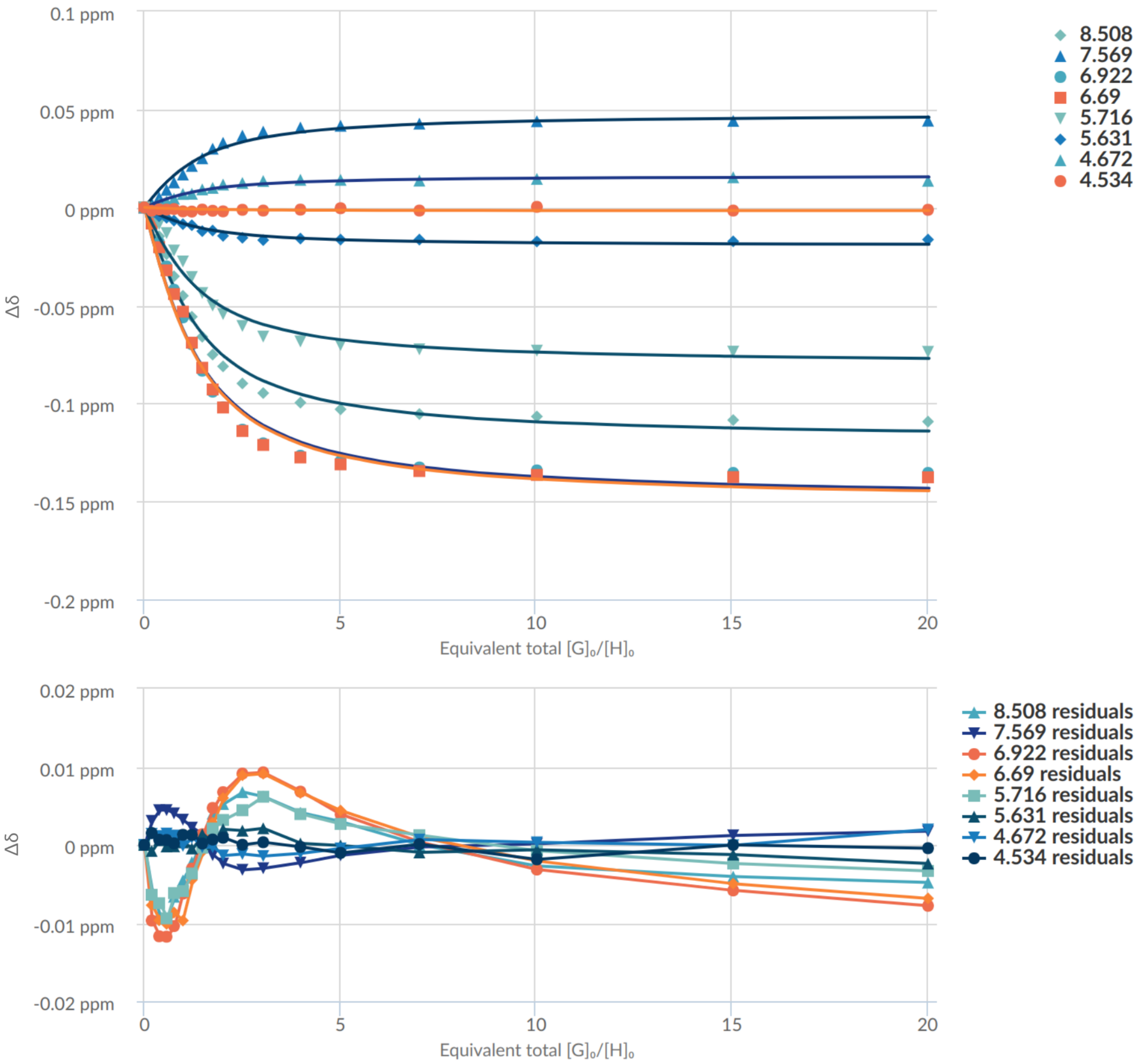

**Parameter (bounds)**	**Optimised**	**Error**	**Initial**
**K** (0 → ∞)	2553.31 M⁻¹	± 5.7772 %	100.00 M⁻¹

Figure S7: Analysis of the titration data from Figure S6 and determination of the binding constant of **AcO**$^-$ with BindFit. The lack of inflection points is an indication for the formation of a 1:1 host–guest complex.

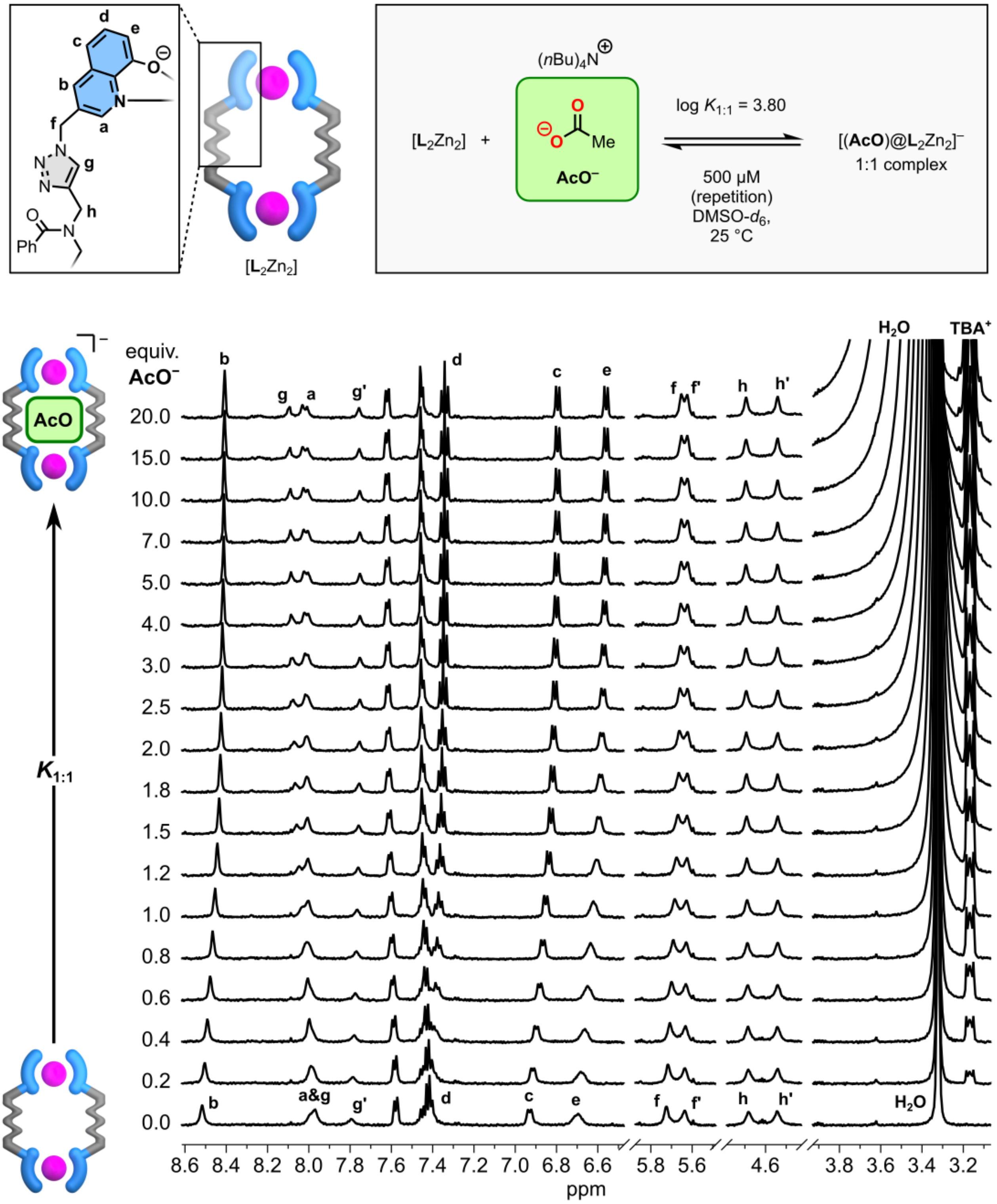

Figure S8: Repetition of the ^{1}H NMR titration of [$\mathbf{L}_2$Zn$_2$] with **AcO**$^-$ as TBA salt (500 MHz, 500 μM, DMSO-d_6, 25 °C).

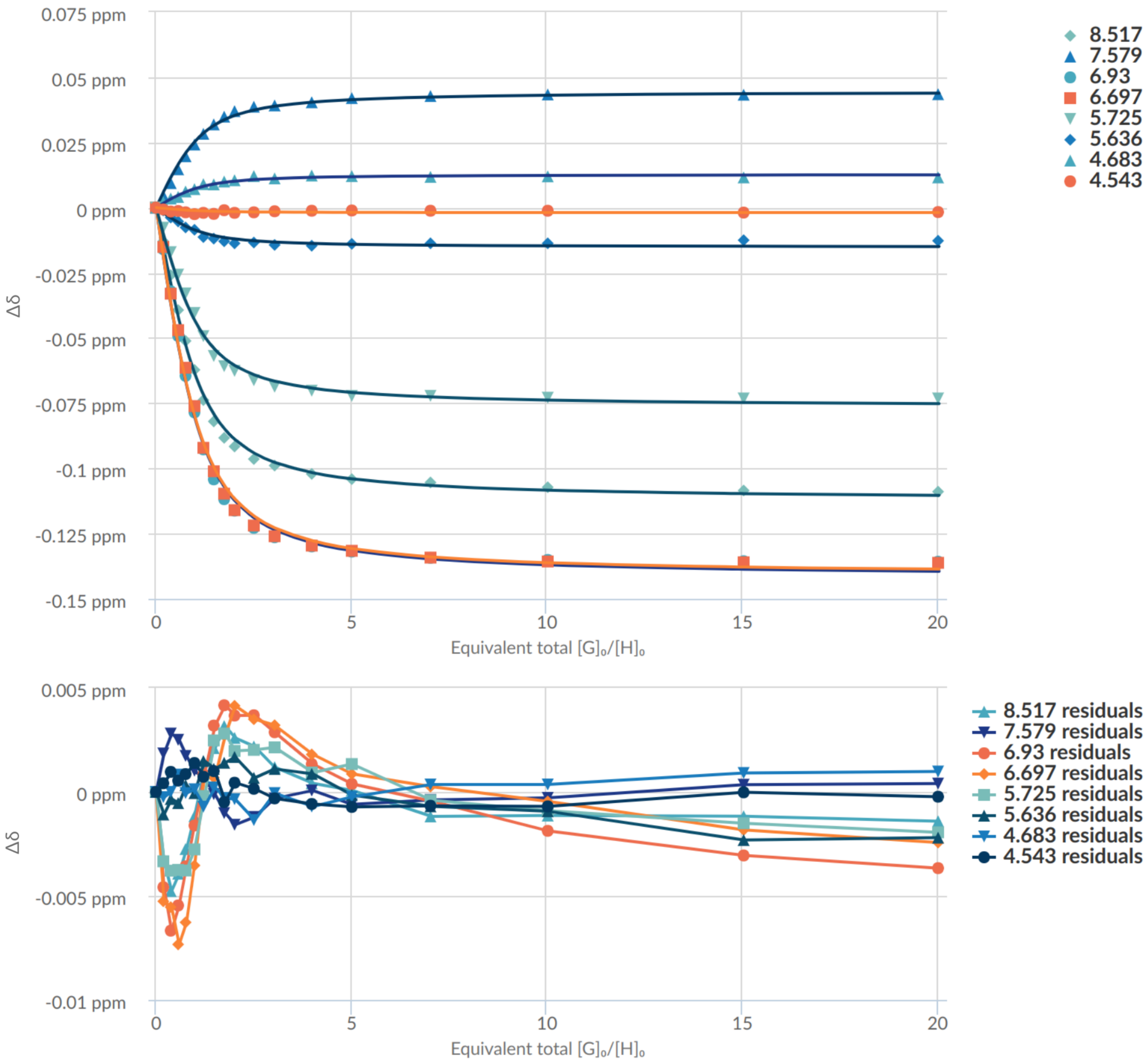

Parameter (bounds)	Optimised	Error	Initial
**K** (0 → ∞)	6257.30 M^{-1}	± 4.0410 %	100.00 M^{-1}

Figure S9: Analysis of the titration data from Figure S8 and determination of the binding constant of **AcO$^-$** with BindFit. Inflection points are not observed.

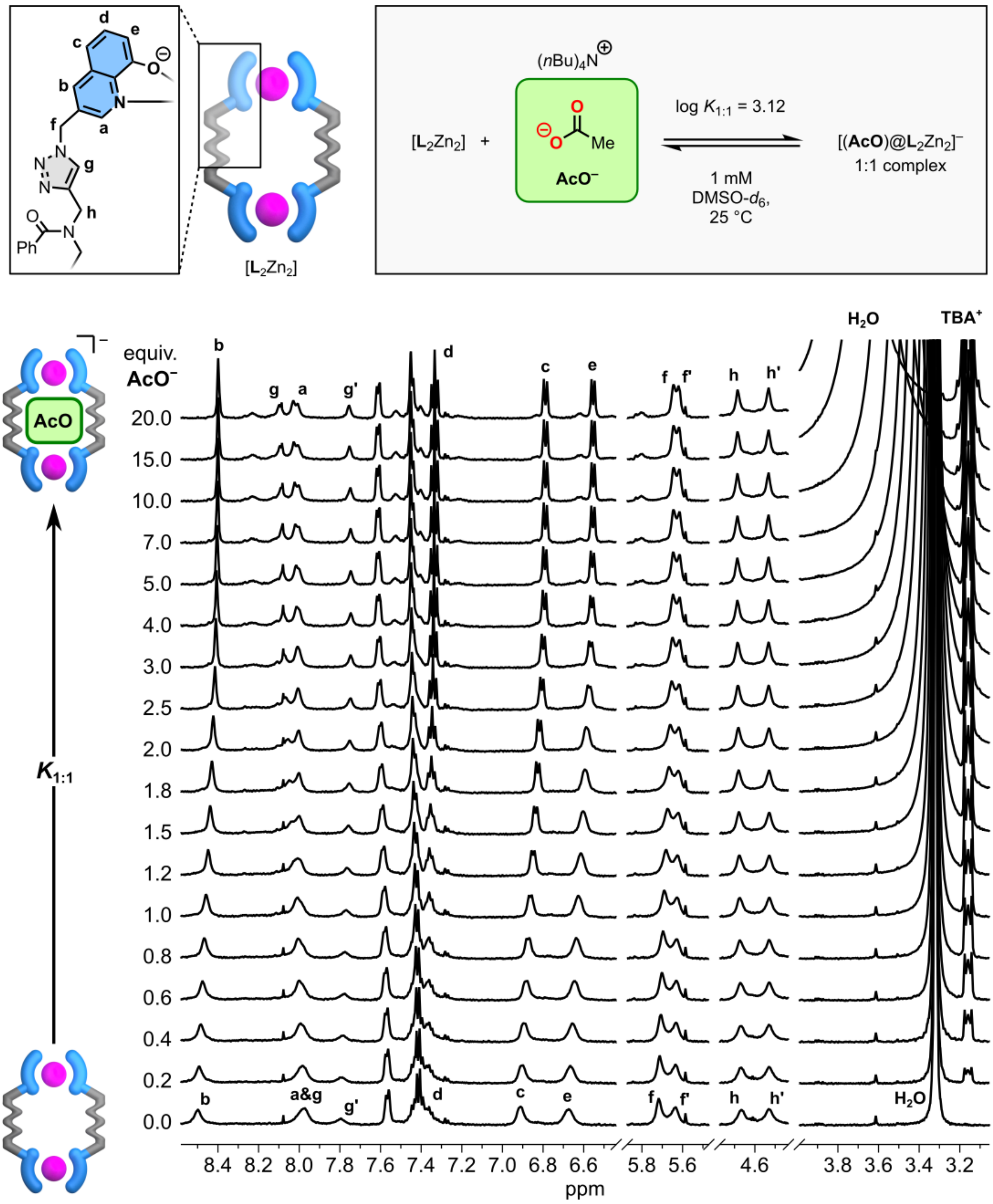

Figure S10: ^{1}H NMR titration ($500\,\mathrm{MHz}$, $1\,\mathrm{mM}$, DMSO-d_6, $25\,^\circ\mathrm{C}$) of [$\mathbf{L}_2Zn_2$] with **AcO**$^-$ as TBA salt.

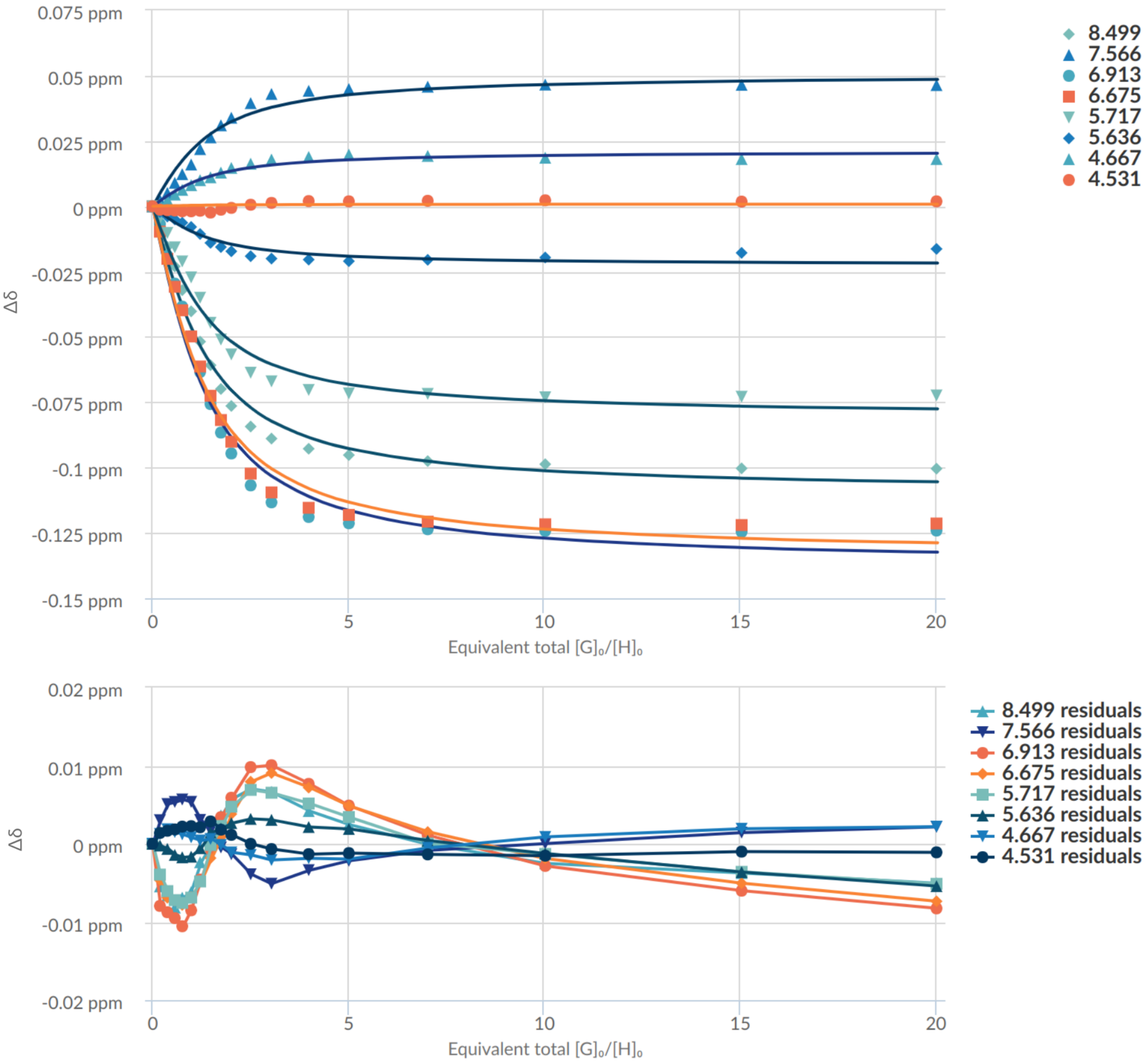

Parameter (bounds)	Optimised	Error	Initial
**K** (0 → ∞)	1315.71 M^{-1}	± 6.2070 %	100.00 M^{-1}

Figure S11: Analysis of the titration data from Figure S10 and determination of the binding constant of **AcO^-** with BindFit. Inflection points are again not observed.

3.3. Benzoate

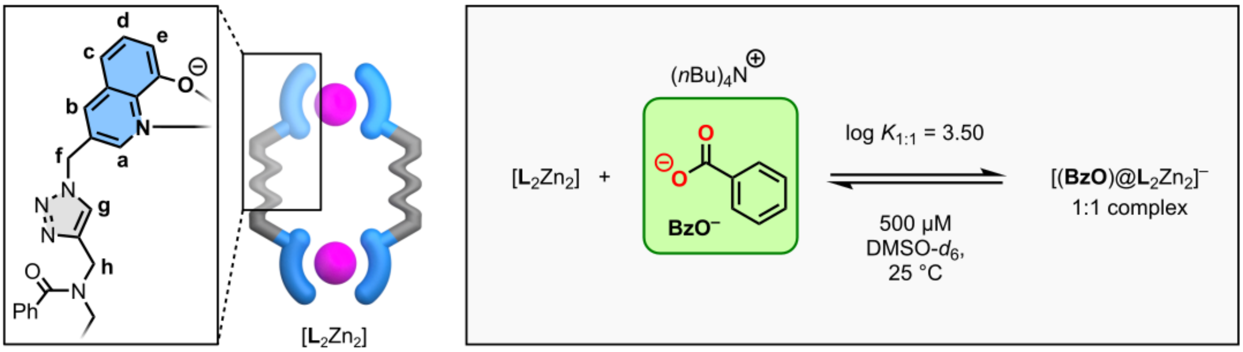

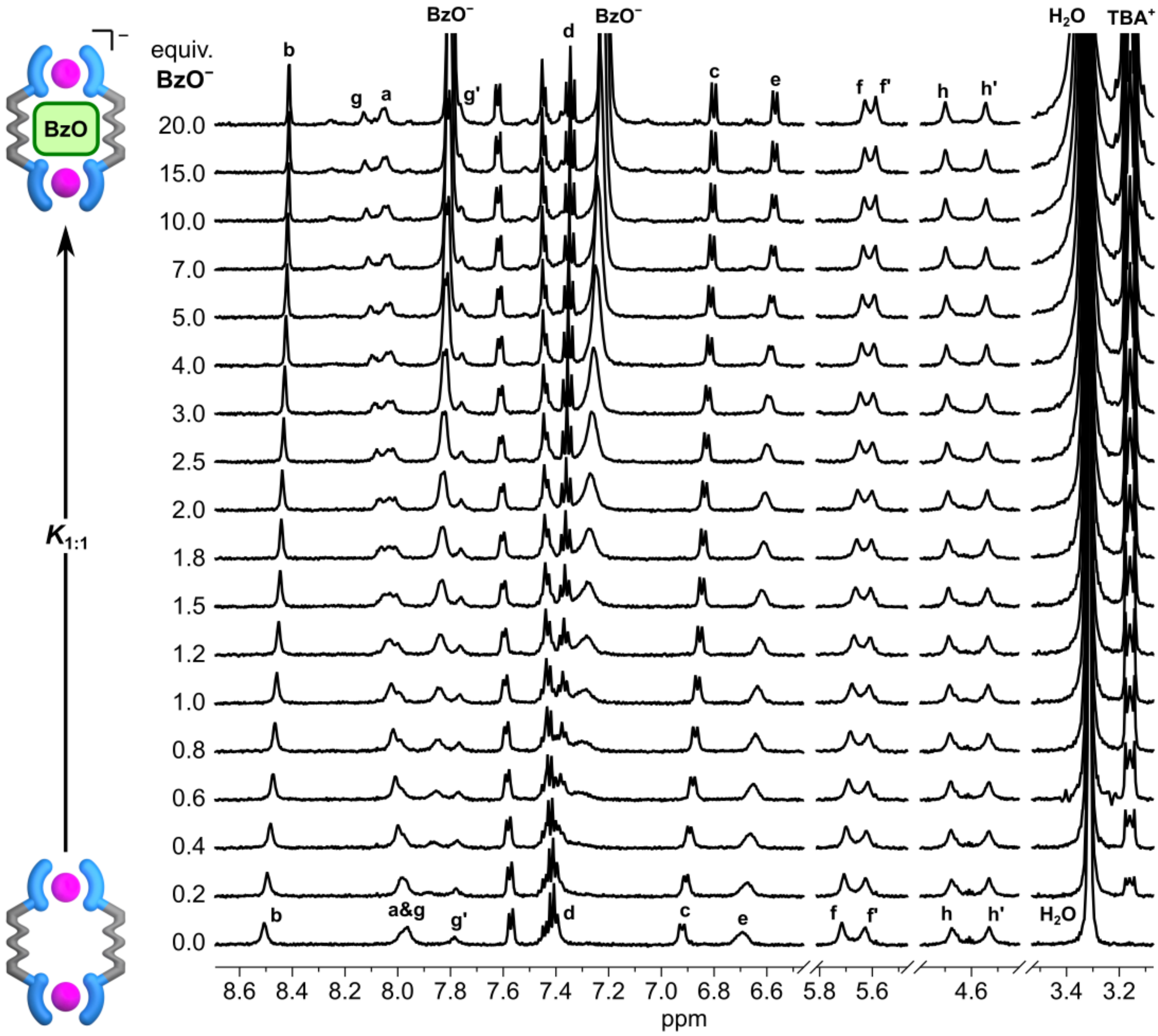

Figure S12: ^{1}H NMR titration (500 MHz, 500 μM, DMSO-d_6, 25 °C) of [$\mathbf{L}_2$Zn$_2$] with tetrabutylammonium benzoate (**BzO**$^-$).

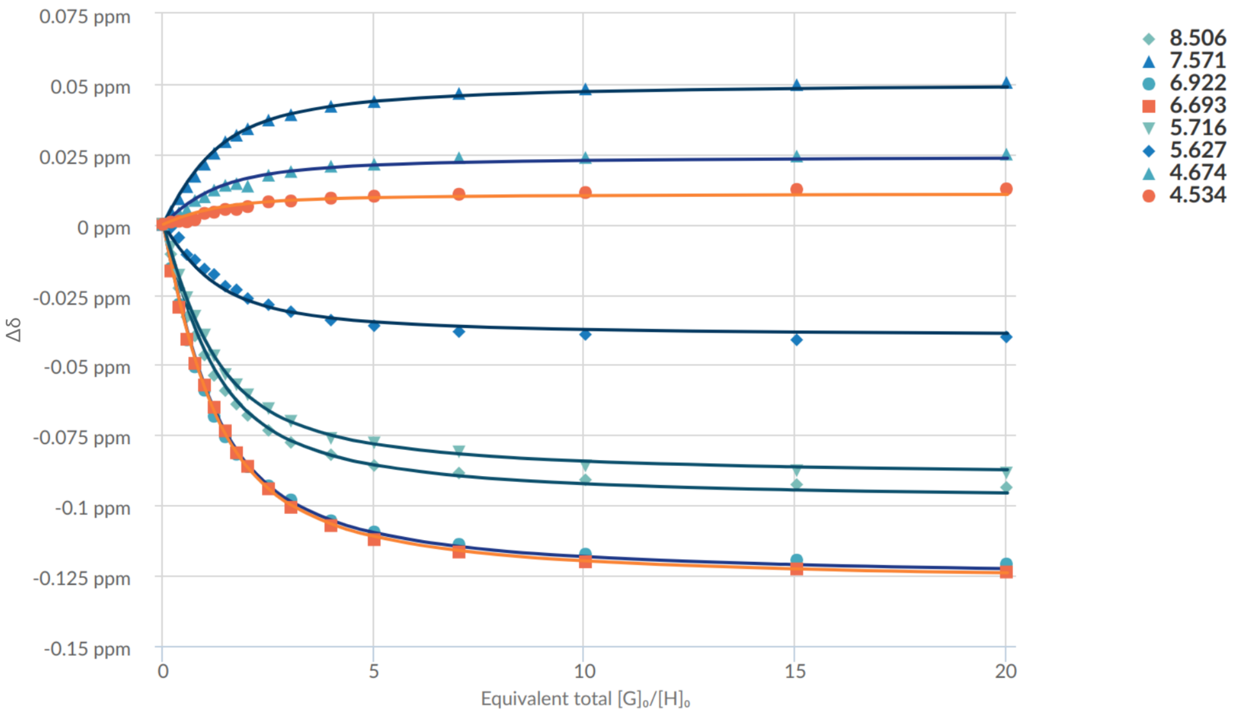

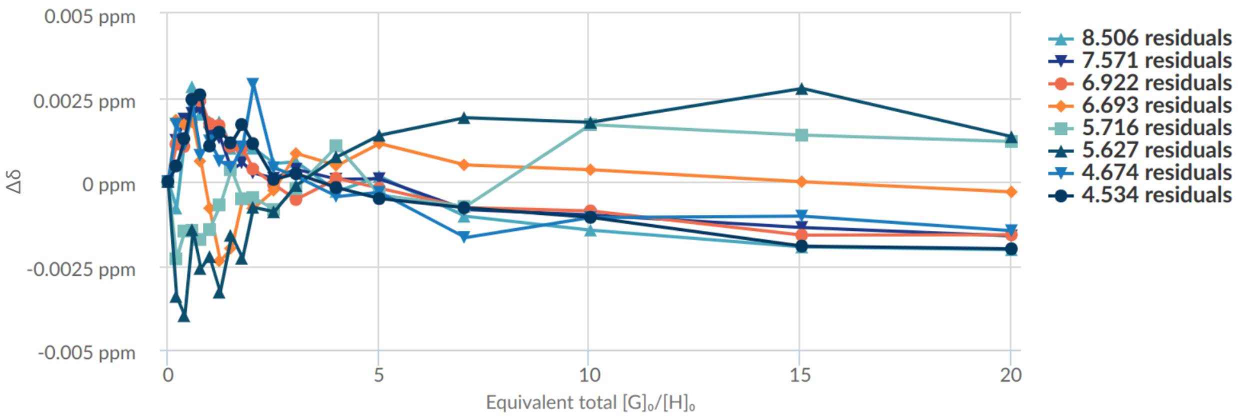

**Parameter (bounds)**	**Optimised**	**Error**	**Initial**
**K** (0 → ∞)	3133.83 M^{-1}	± 2.1519 %	100.00 M^{-1}

Figure S13: Analysis of the titration data from Figure S12 and determination of the binding constant of **BzO**$^-$ with BindFit. The lack of inflection points is an indication for the formation of a 1:1 host–guest complex.

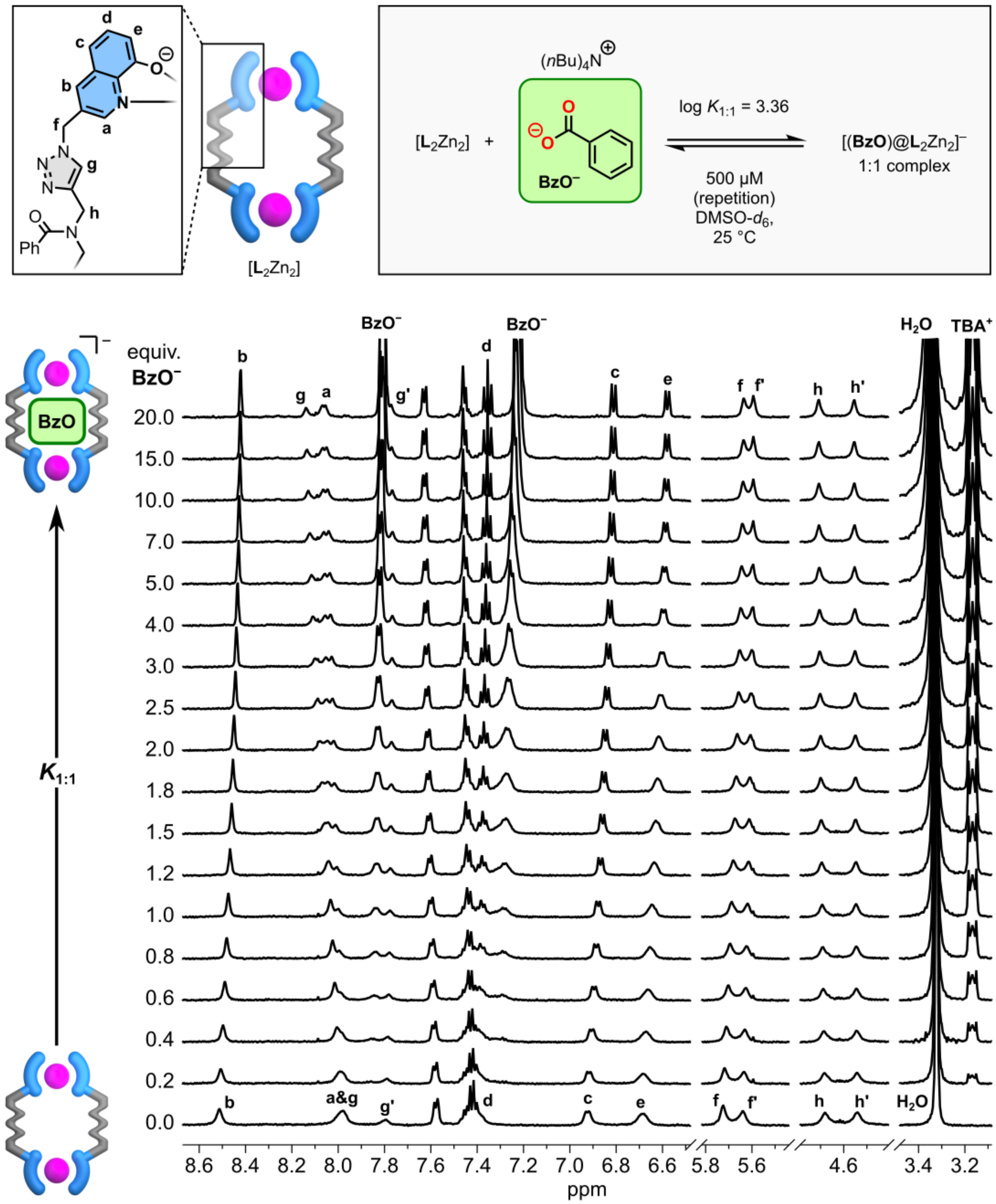

Figure S14: Repetition of the ^{1}H NMR titration of [$\mathbf{L}_2$Zn$_2$] with **BzO**$^-$ as TBA salt (500 MHz, 500 μM, DMSO-d_6, 25 °C).

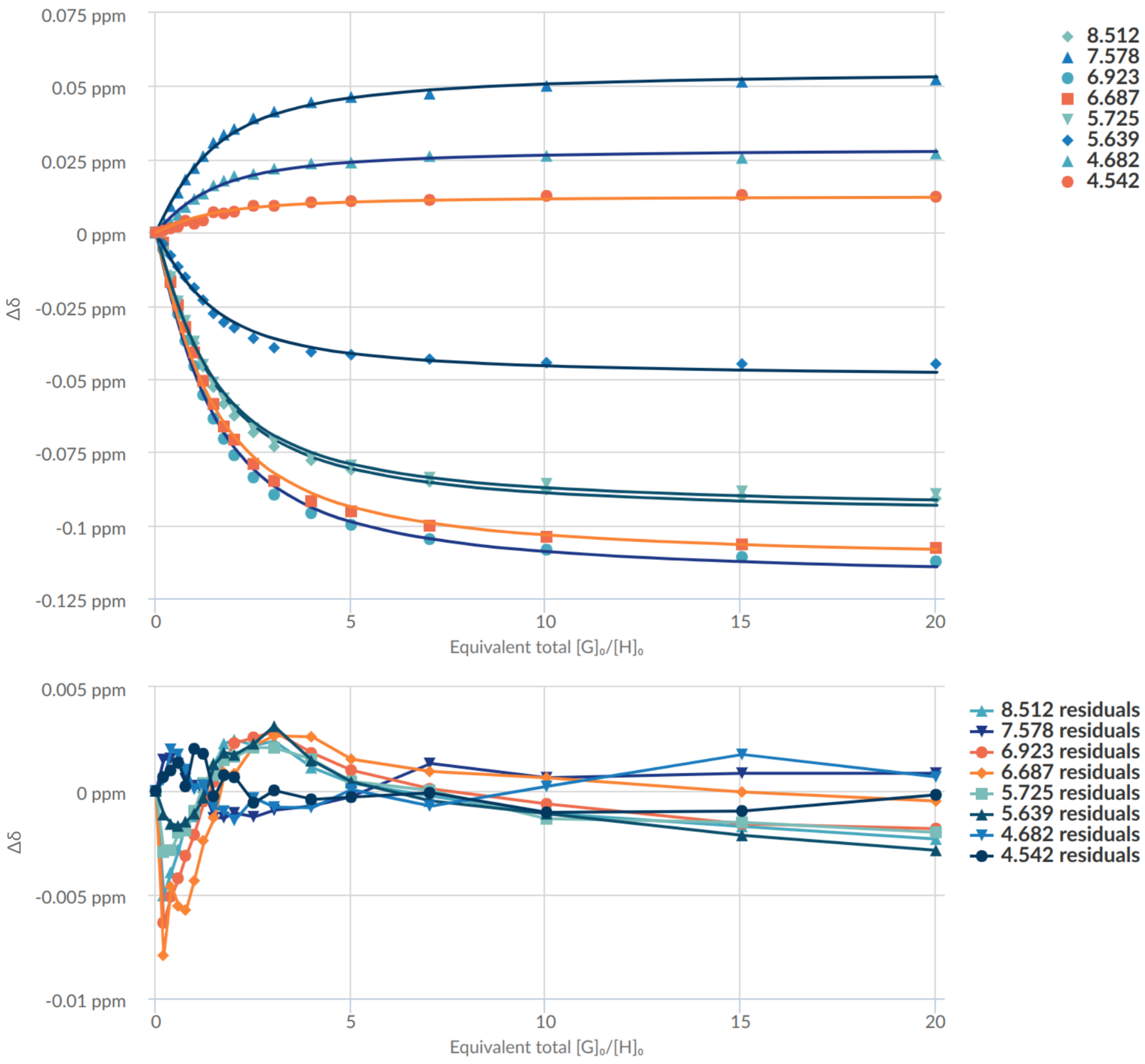

Parameter (bounds)	Optimised	Error	Initial
**K** (0 → ∞)	2304.49 M^{-1}	± 2.9269 %	100.00 M^{-1}

Figure S15: Analysis of the titration data from Figure S14 and determination of the binding constant of **BzO⁻** with BindFit. Inflection points are not observed.

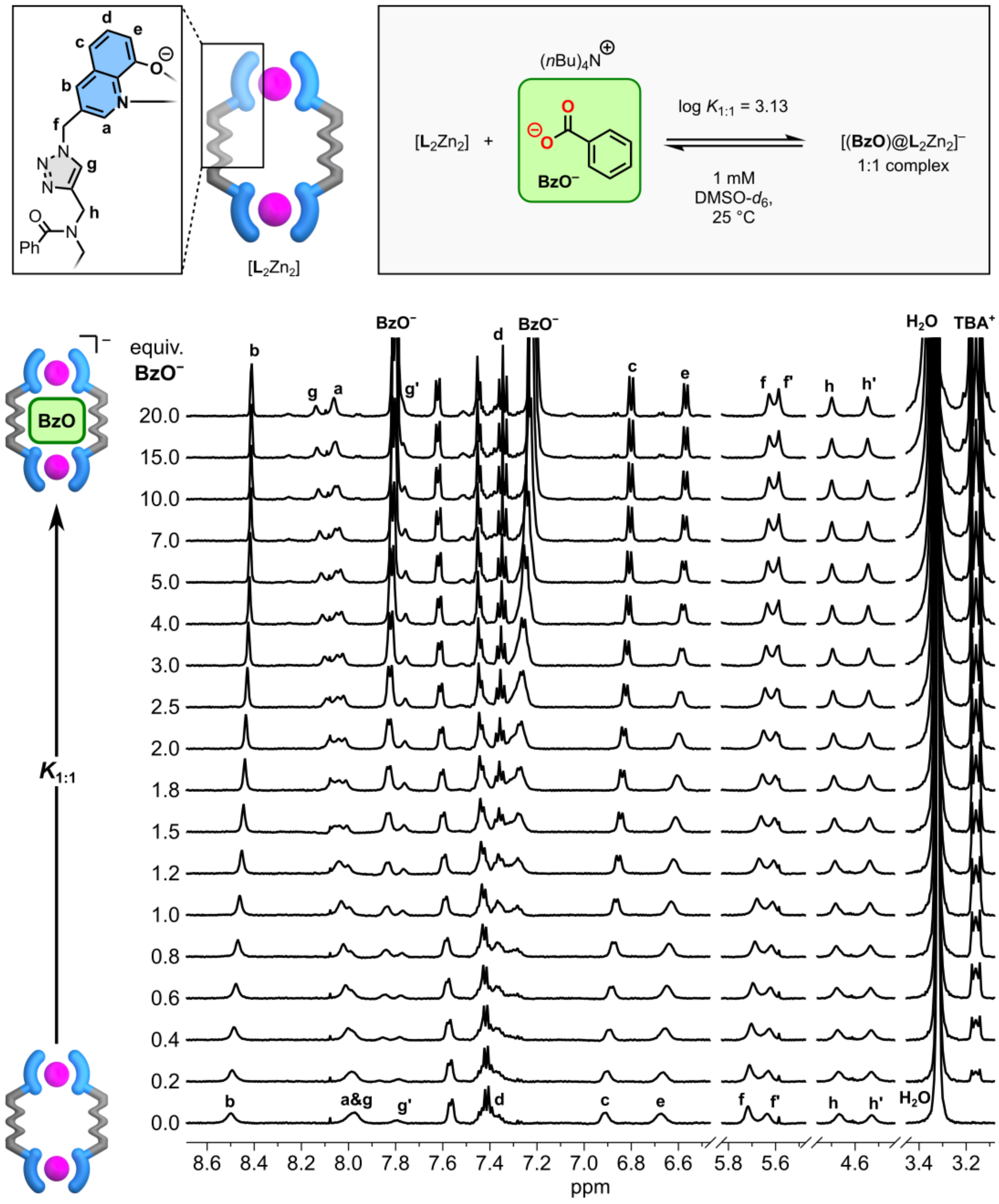

Figure S16: ^{1}H NMR titration (500 MHz, 1 mM, DMSO-d_6, 25 °C) of [**L**$_2$Zn$_2$] with **BzO**$^-$ as TBA salt.

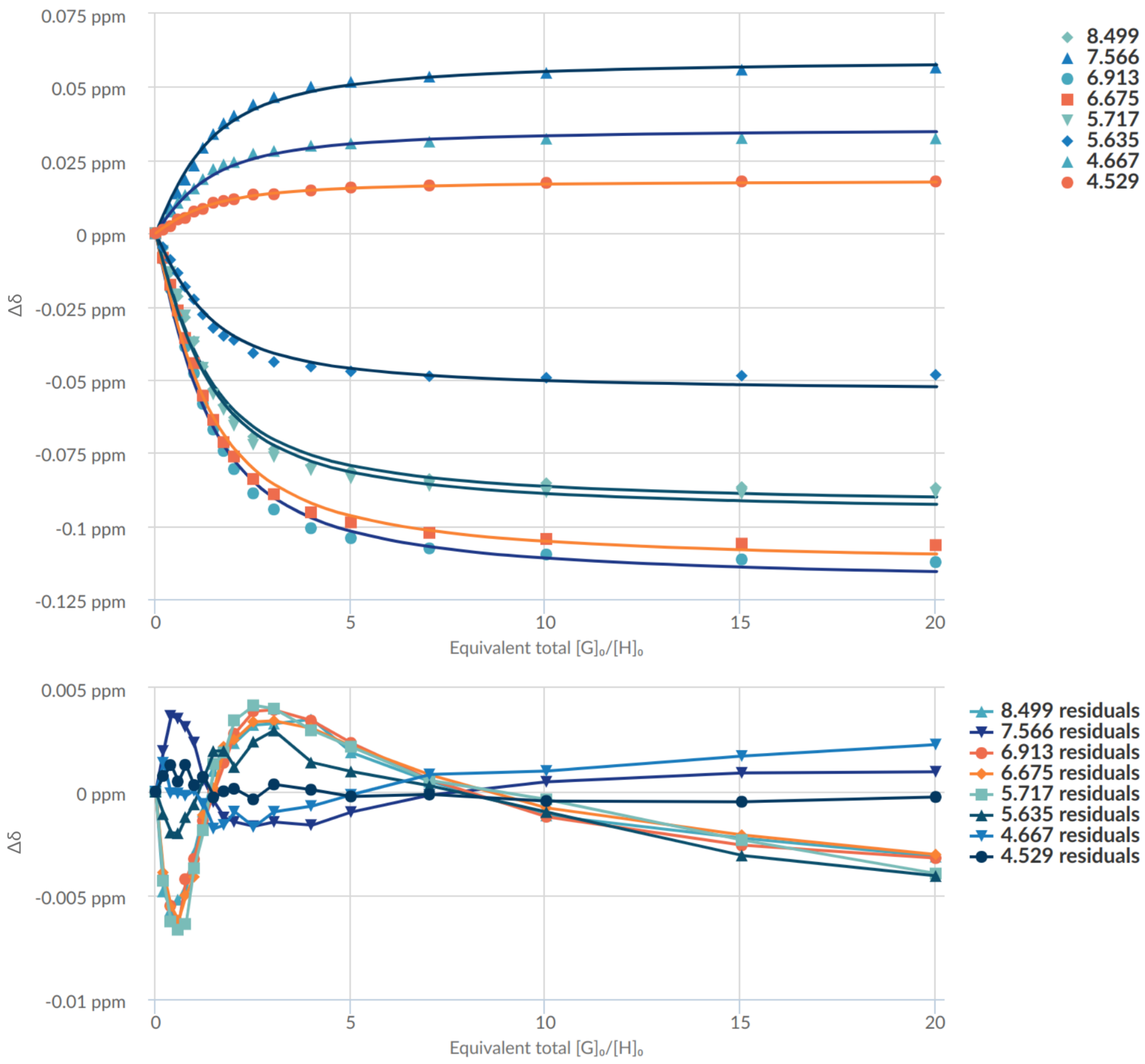

Parameter (bounds)	Optimised	Error	Initial
**K** (0 → ∞)	1337.73 M^{-1}	± 3.8996 %	100.00 M^{-1}

Figure S17: Analysis of the titration data from Figure S16 and determination of the binding constant of BzO^- with BindFit. Inflection points are again not observed.

3.4. Oxalate

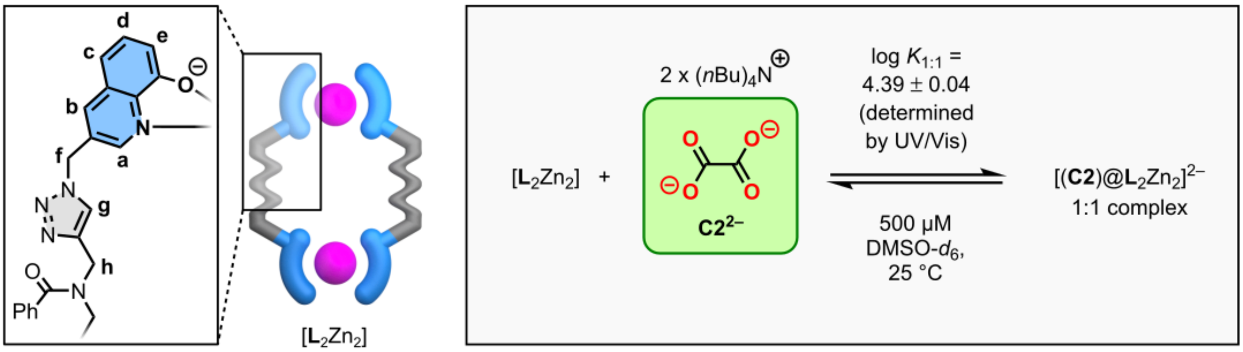

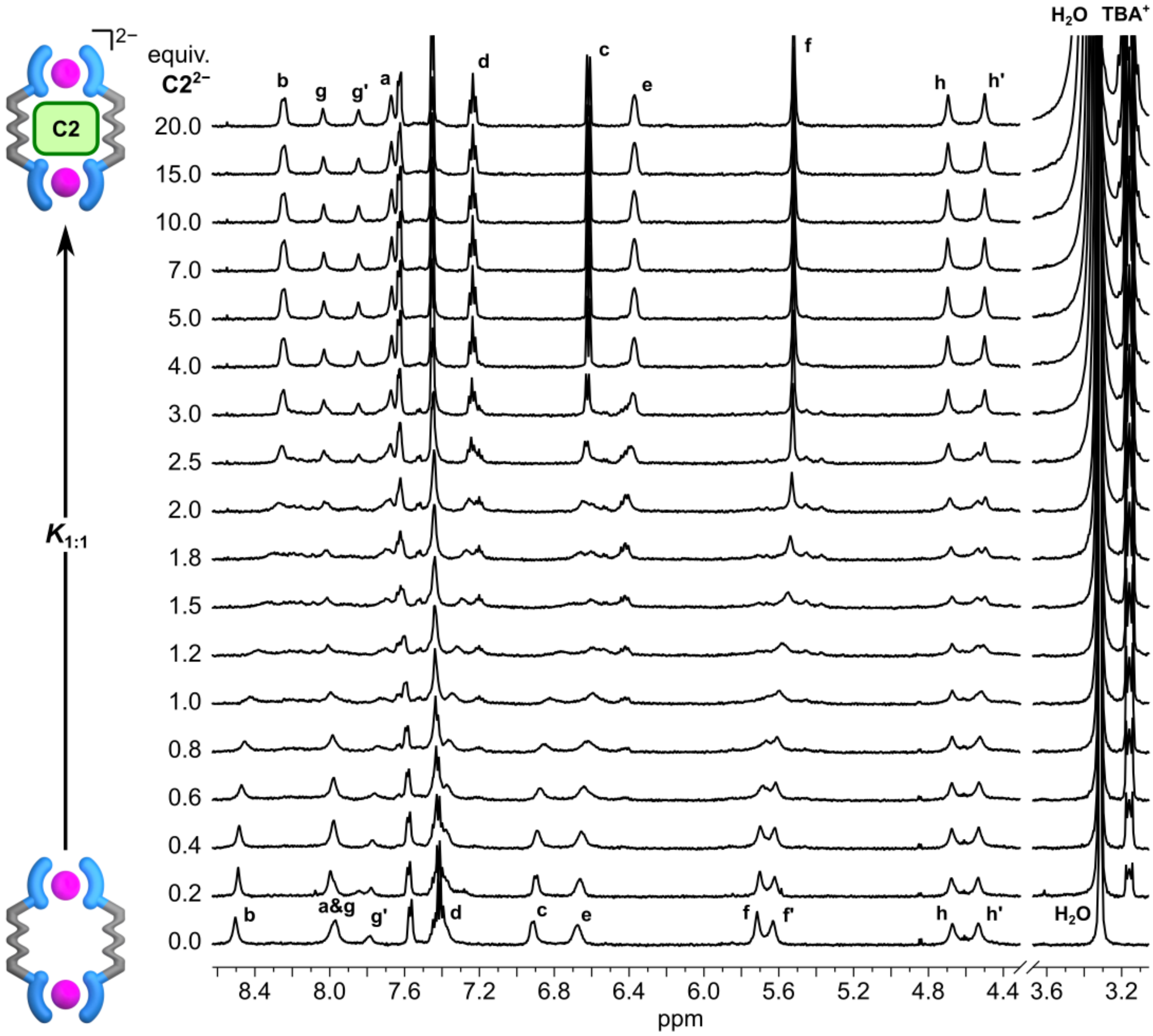

Figure S18: ^{1}H NMR titration (500 MHz, 500 µM, DMSO-d_6, 25 °C) of [L_2Zn_2] with tetrabutylammonium oxalate (**C2**$^{2-}$). Broadening of the signals is observed between one and two equivalents of added guest. The intermediate-like exchange hinders an accurate determination of the binding constants and because of that, UV–vis spectroscopy was used for this task.

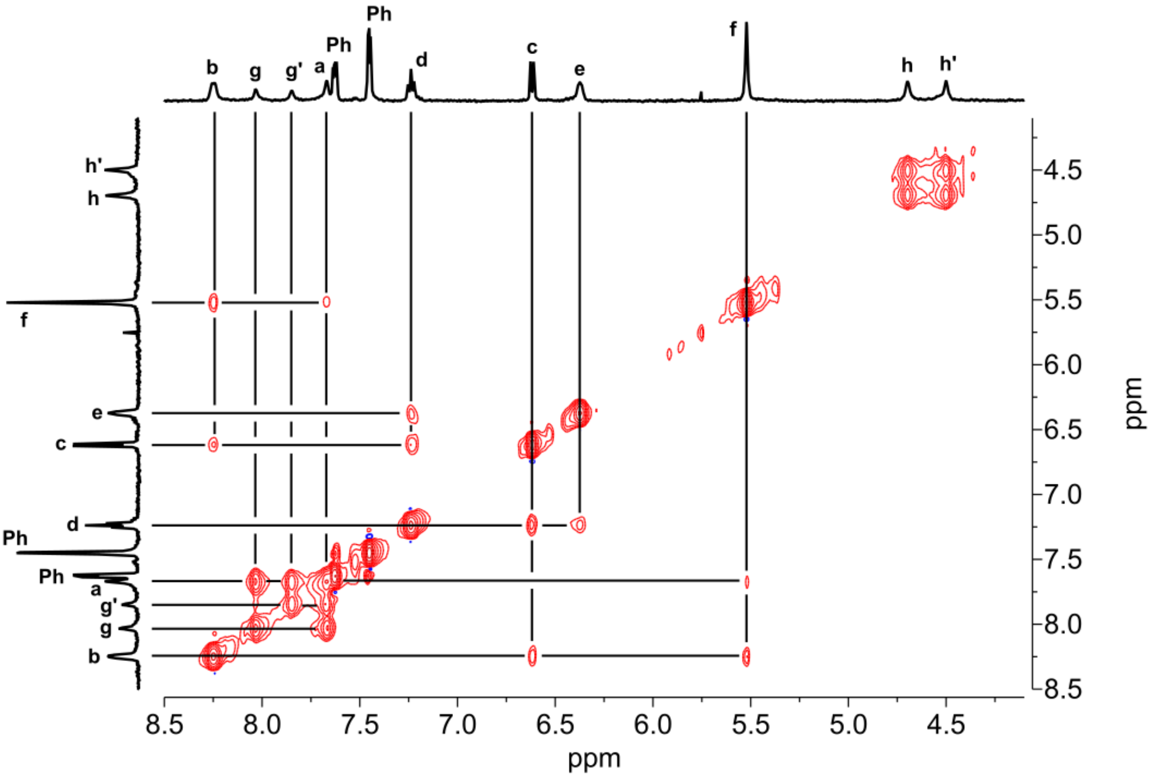

Figure S19: NOESY NMR spectrum (500 MHz, 500 µM, DMSO-d_6, 25 °C) of [(**C2**)@**L**$_2$Zn$_2$]$^{2-}$. The host-guest complex was formed by the addition of 5 equiv. oxalate (TBA salt) to [**L**$_2$Zn$_2$].

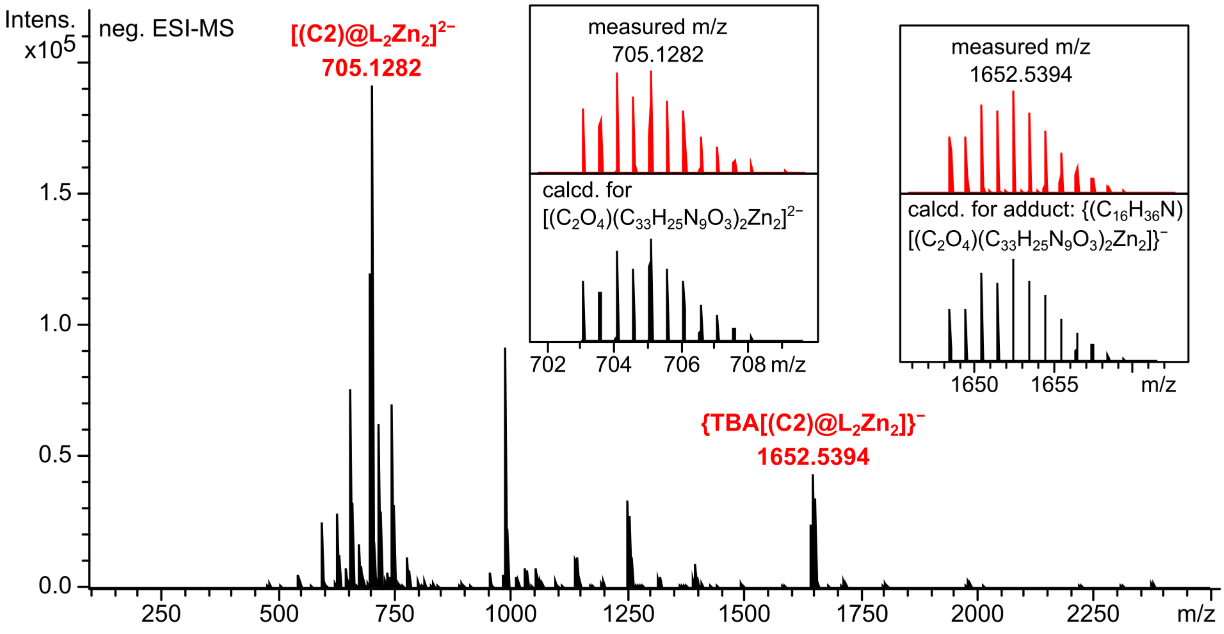

Figure S20: ESI-MS spectrum of [**L**$_2$Zn$_2$] with 5 equiv oxalate as TBA salt. Host and guest were mixed in DMSO before dilution and injection.

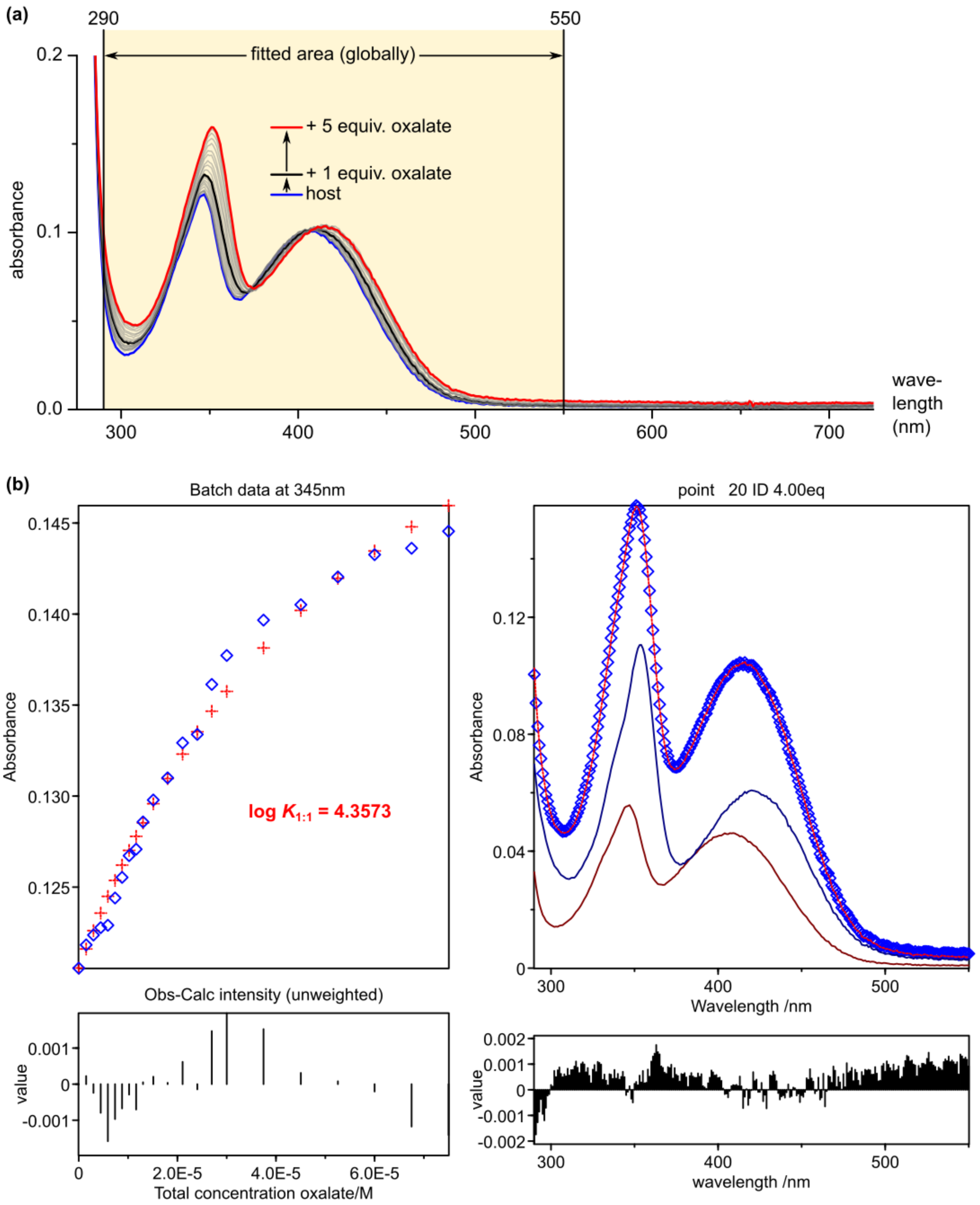

Figure S21: a) UV–vis titration ($15\,\mu M$, $l = 1\,cm$, DMSO-d_6) of [L_2Zn_2] with oxalate (**C2**$^{2-}$) as TBA salt. b) HypSpec2014 result of fitting the absorbance data in the area between $290\,nm$ and $550\,nm$ by applying a 1:1 binding model.

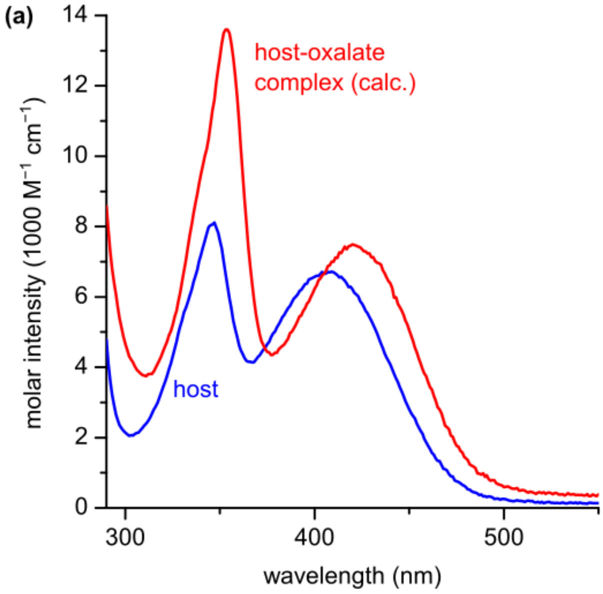

(b)

HypSpec refinement output. Version number 1.2.35

Converged in 5 iterations with sigma = 1.2198E-03

Log beta	value	standard deviation
(L2Zn2)oxalate		
	4.3573	0.0074

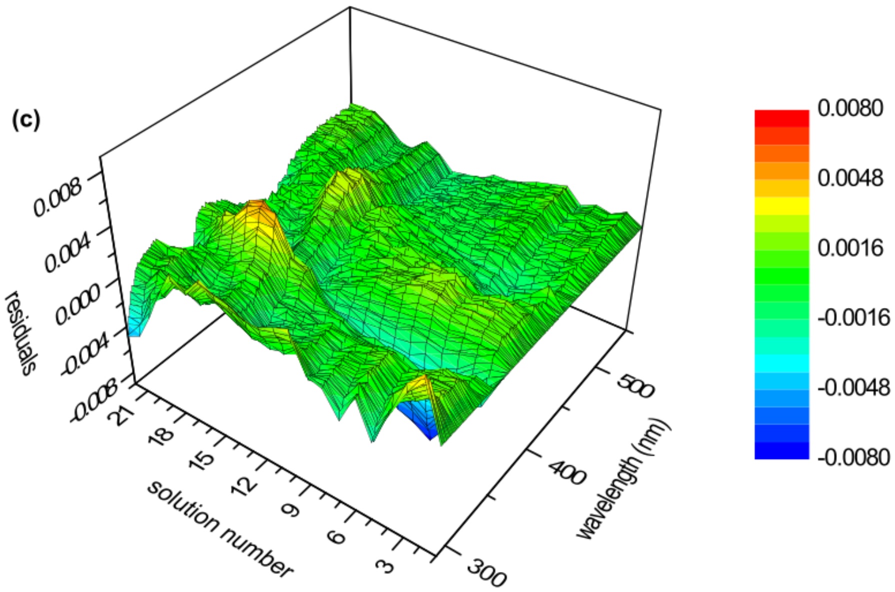

Figure S22: Additional information about the fitting result shown in Figure S21. a) Molar intensity of the involved absorbing species. b) Information about the fitting process. c) Residuals map = Difference between measured and calculated (fitted) data.

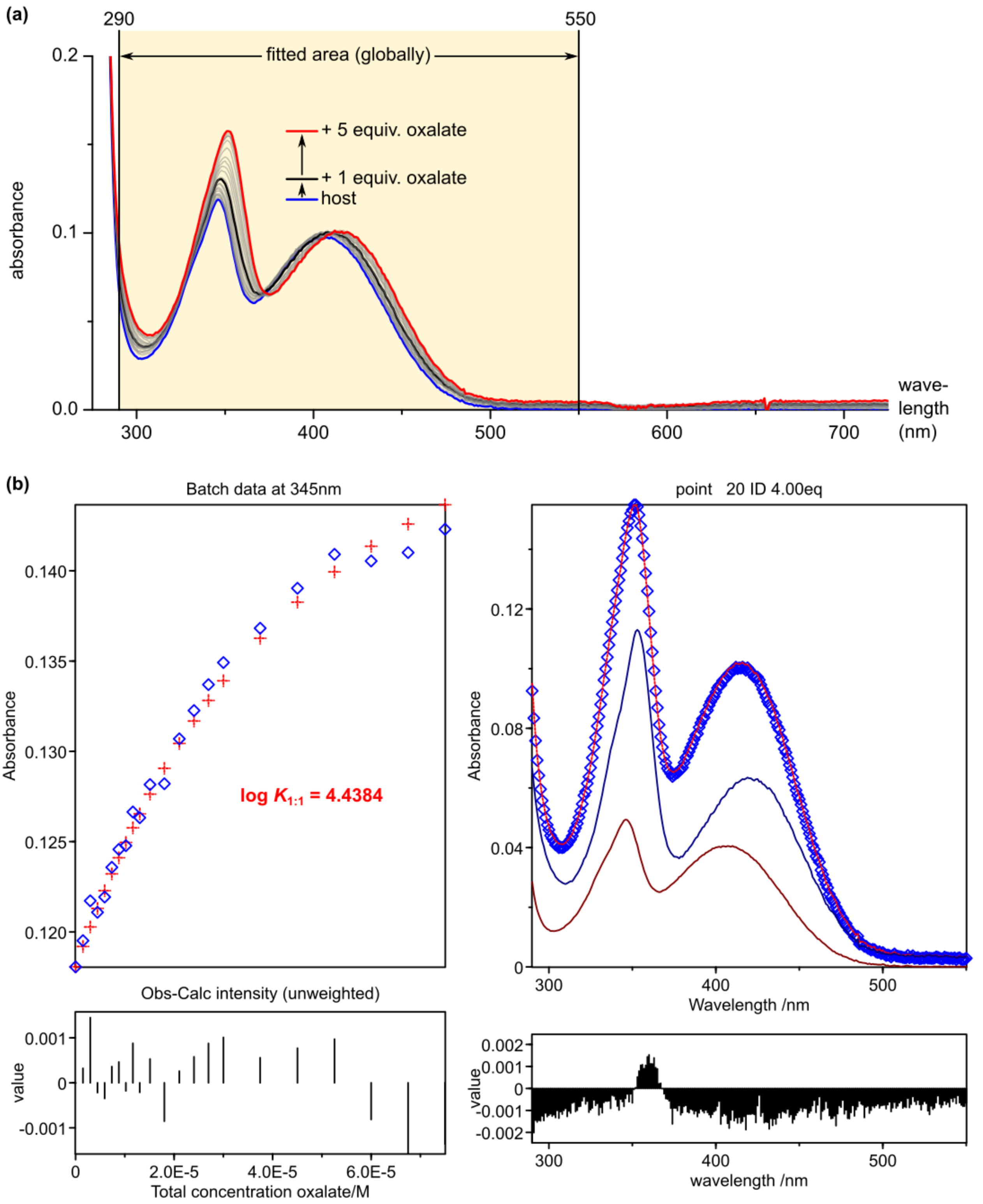

Figure S23: a) Repeated UV–vis titration ($15\,\mu\text{M}$, $l = 1\,\text{cm}$, DMSO-d_6) of [$\mathbf{L}_2$Zn$_2$] with oxalate (**C2**$^{2-}$) as TBA salt. b) HypSpec2014 result of fitting the absorbance data in the area between $290\,\text{nm}$ and $550\,\text{nm}$ by applying a 1:1 binding model.

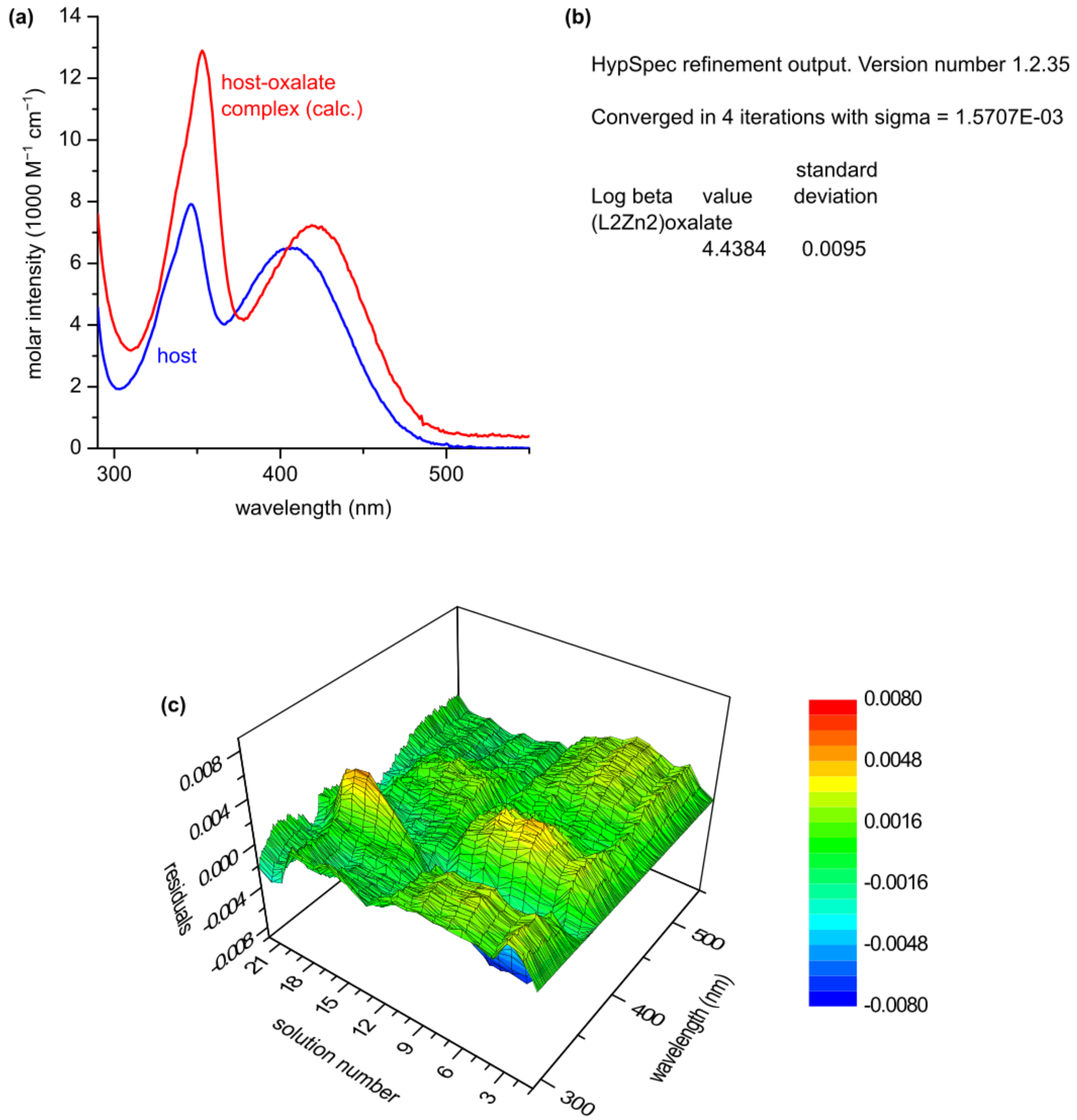

Figure S24: Additional information about the fitting result shown in Figure S23. a) Molar intensity of the involved absorbing species. b) Information about the fitting process. c) Residuals map = Difference between measured and calculated (fitted) data.

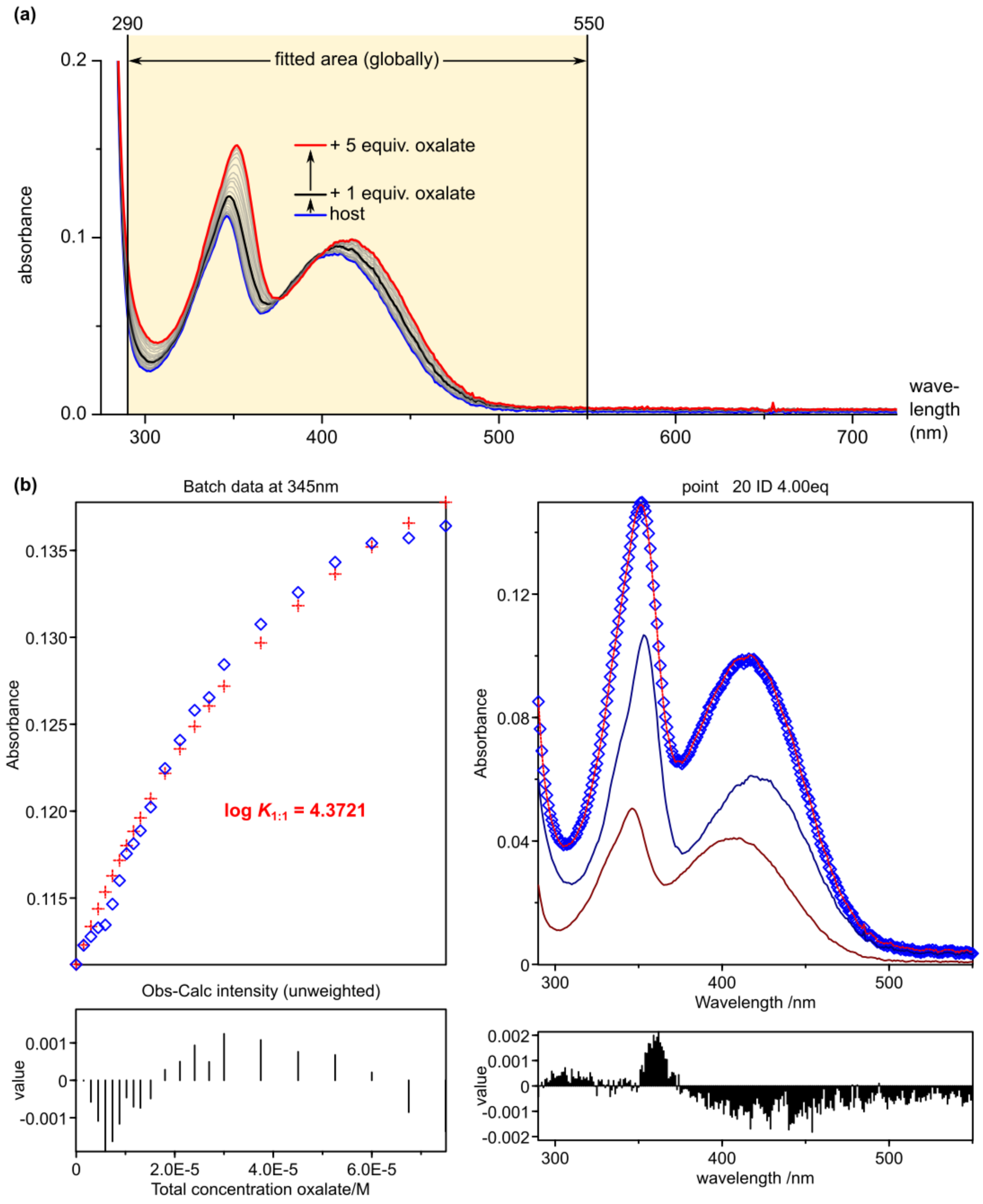

Figure S25: Repeated UV–vis titration ($15\,\mu M$, $l = 1\,cm$, DMSO-d_6) of [L_2Zn_2] with oxalate (**C2**$^{2-}$) as TBA salt. b) HypSpec2014 result of fitting the absorbance data in the area between $290\,nm$ and $550\,nm$ by applying a 1:1 binding model.

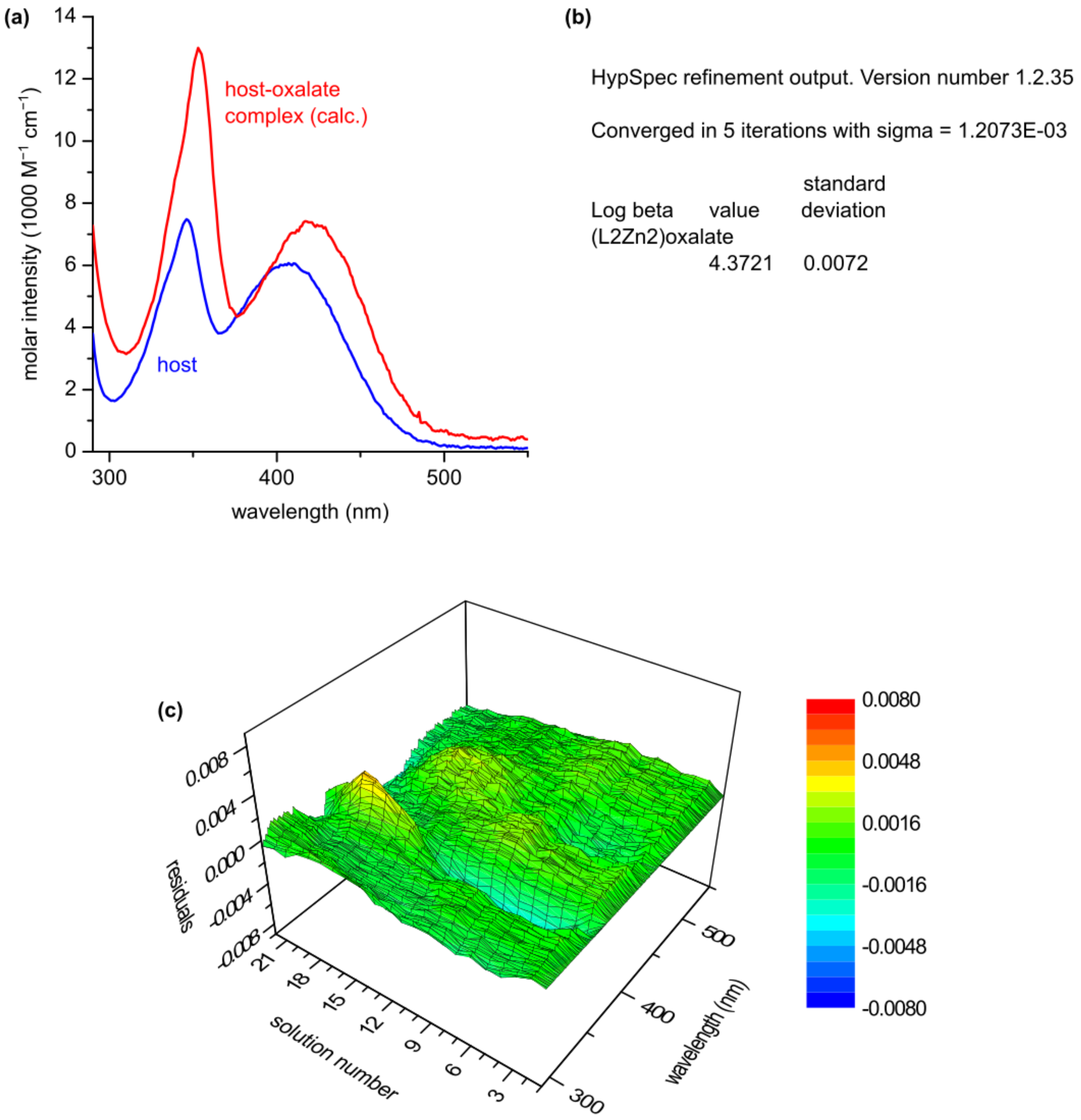

Figure S26: Additional information about the fitting result shown in Figure S25. a) Molar intensity of the involved absorbing species. b) Information about the fitting process. c) Residuals map = Difference between measured and calculated (fitted) data.

3.5. Malonate

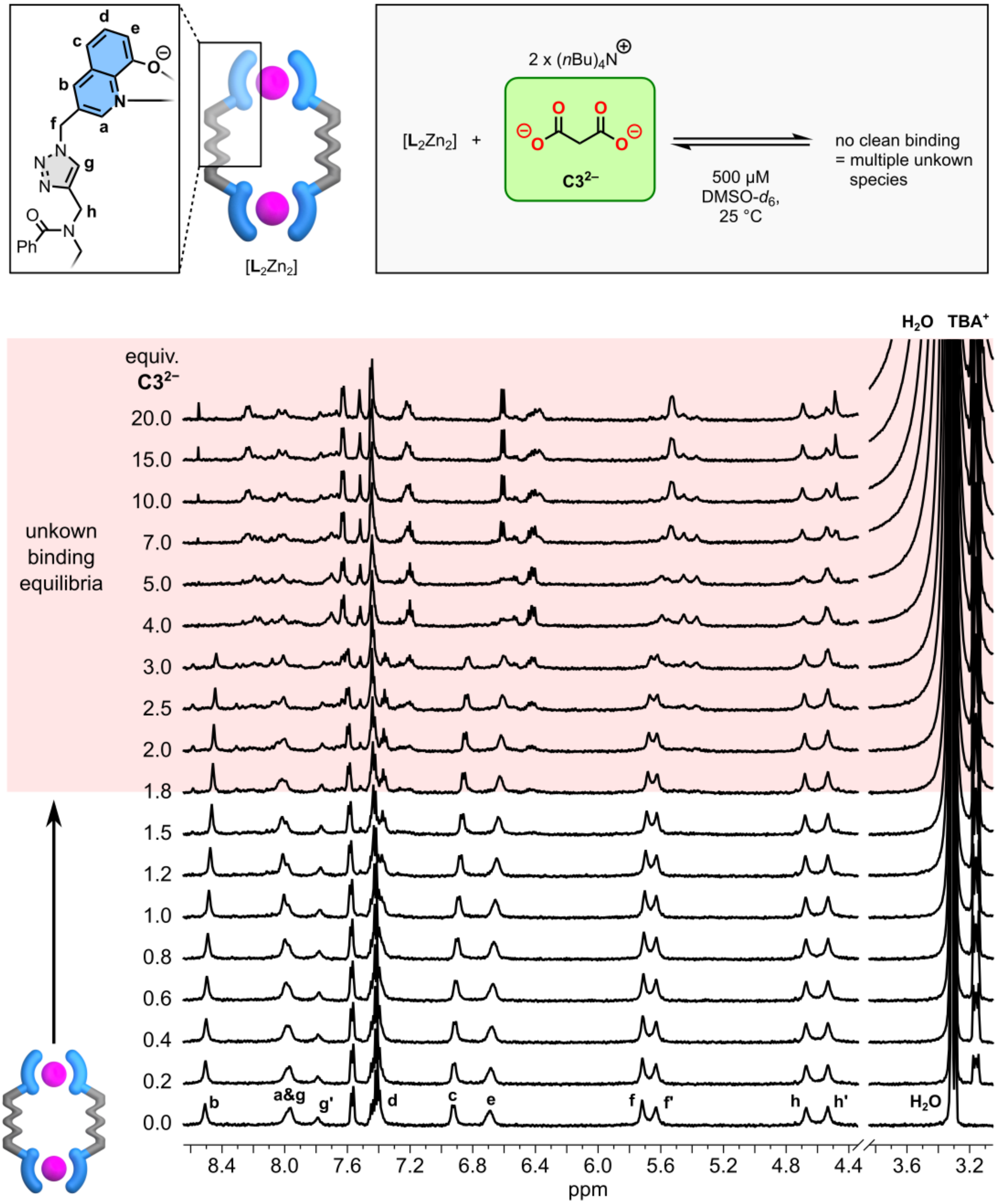

Figure S27: ^{1}H NMR titration (500 MHz, 500 µM, DMSO-d_6, 25 °C) of [$\mathbf{L}_2$Zn$_2$] with tetrabutylammonium malonate (**C3**$^{2-}$).

3.6. Succinate

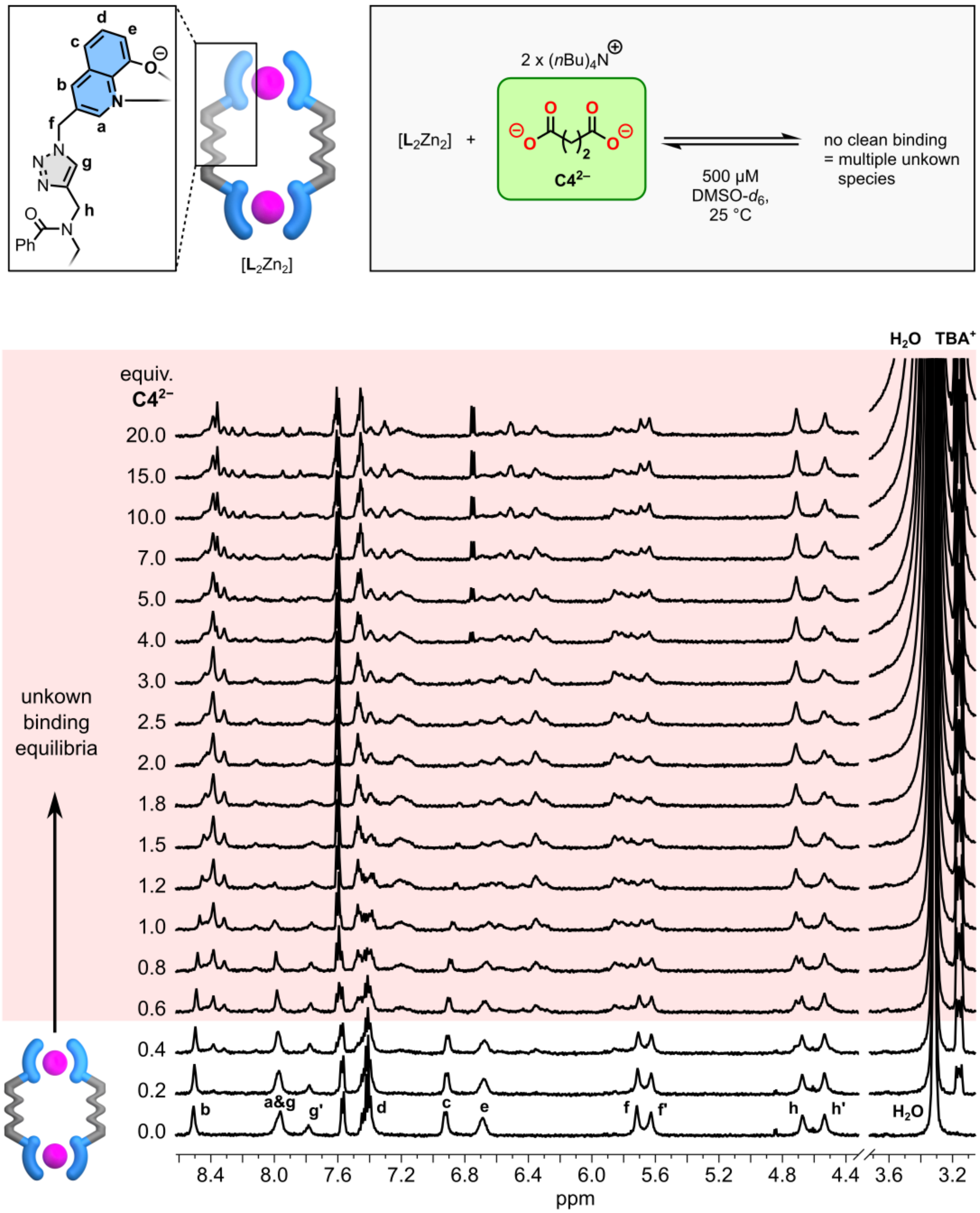

Figure S28: ^{1}H NMR titration (500 MHz, 500 µM, DMSO-d_6, 25 °C) of [L_2Zn_2] with tetrabutylammonium succinate (**C4^{2-}**).

3.7. Glutarate

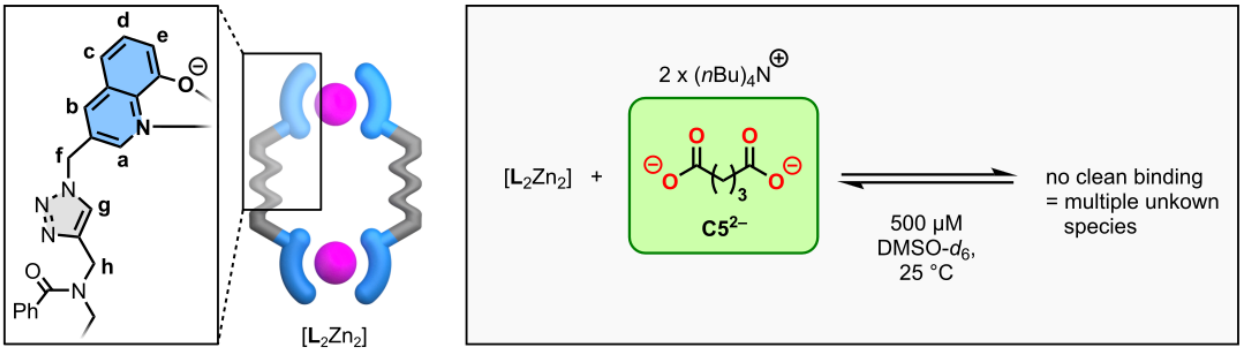

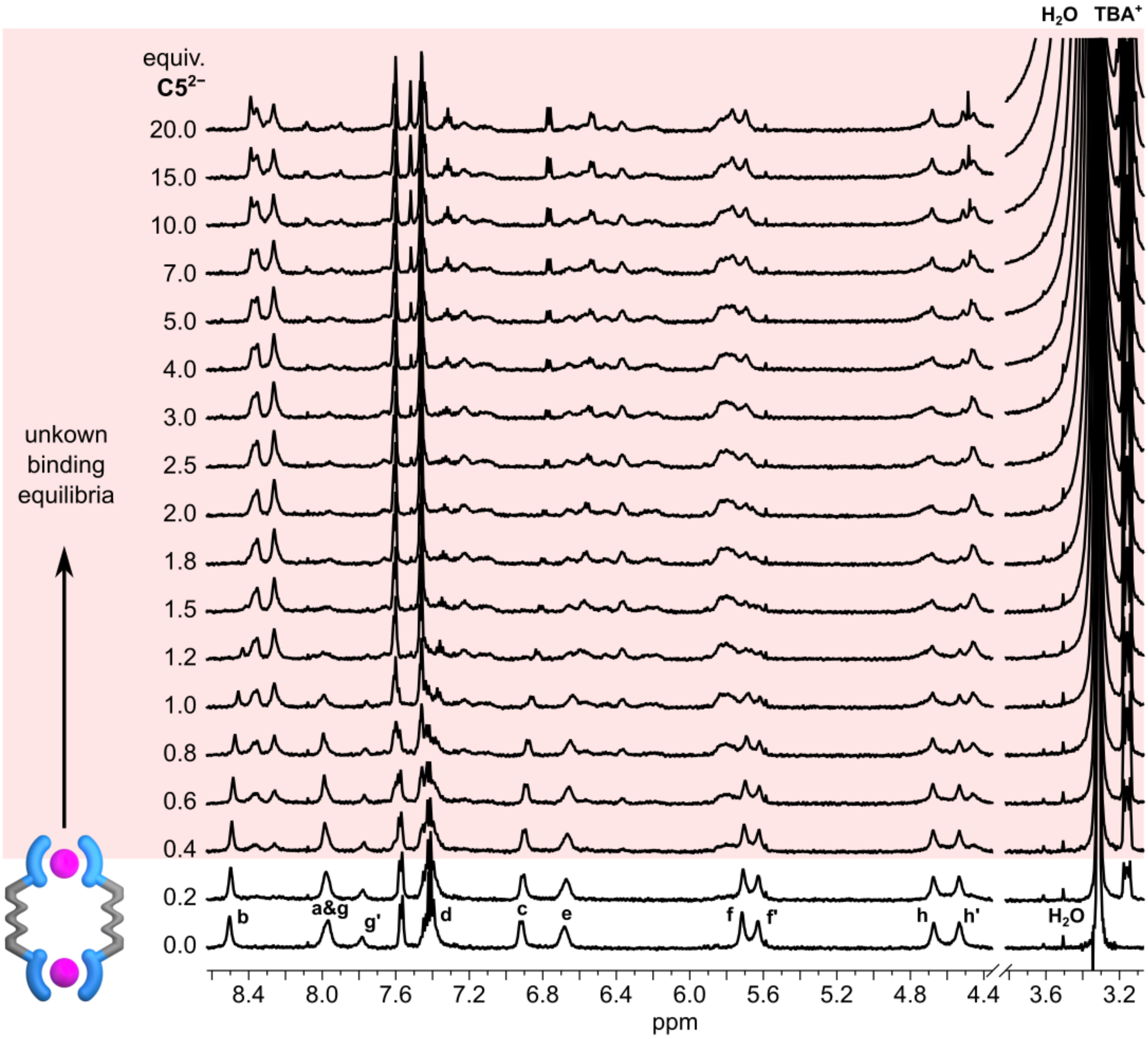

Figure S29: ^{1}H NMR titration (500 MHz, 500 µM, DMSO-d_6, 25 °C) of [$\mathbf{L}_2$Zn$_2$] with tetrabutylammonium glutarate (**C5^{2-}**).

3.8. Adipate

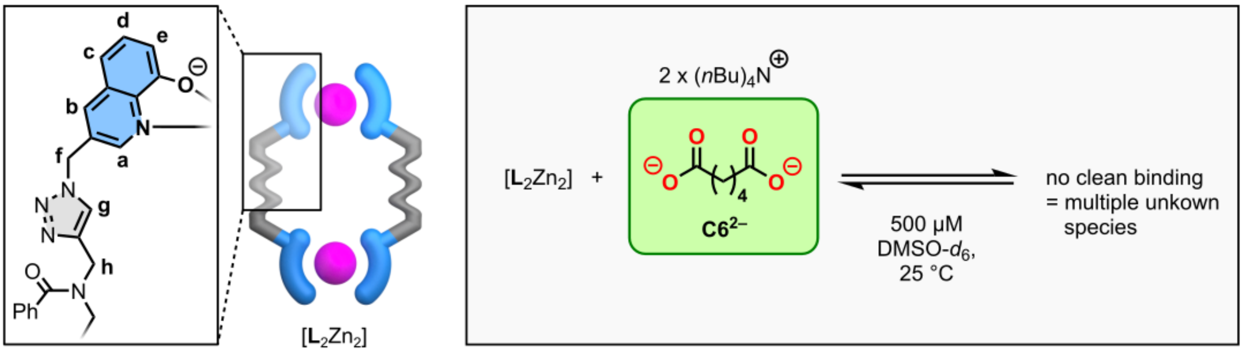

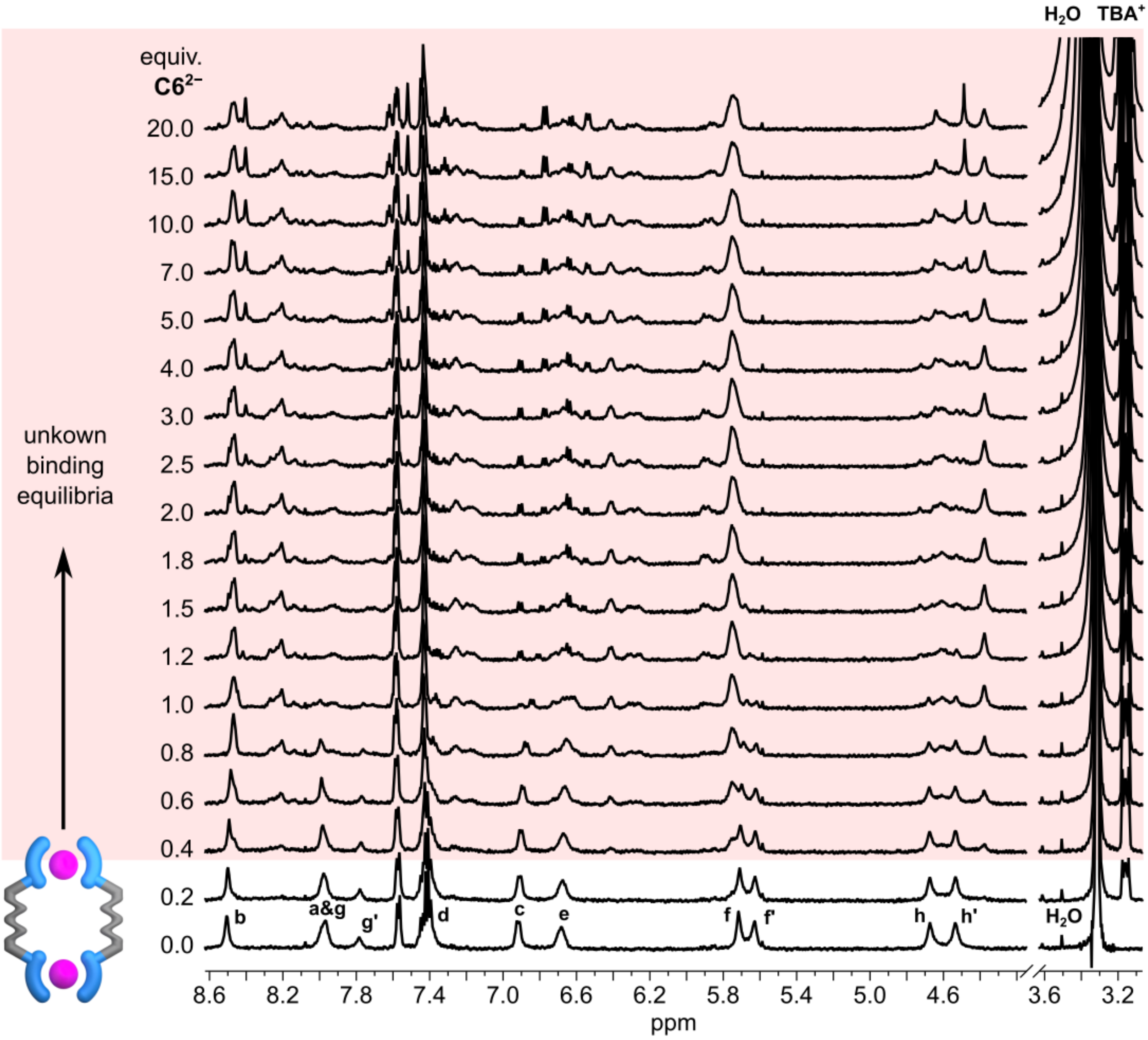

Figure S30: ^{1}H NMR titration ($500\,\mathrm{MHz}$, $500\,\mathrm{\mu M}$, DMSO-d_6, $25\,°\mathrm{C}$) of [$\mathbf{L}_2Zn_2$] with tetrabutylammonium adipate (**C6^{2-}**).

3.9. Nitrate, sulfate and dithionite

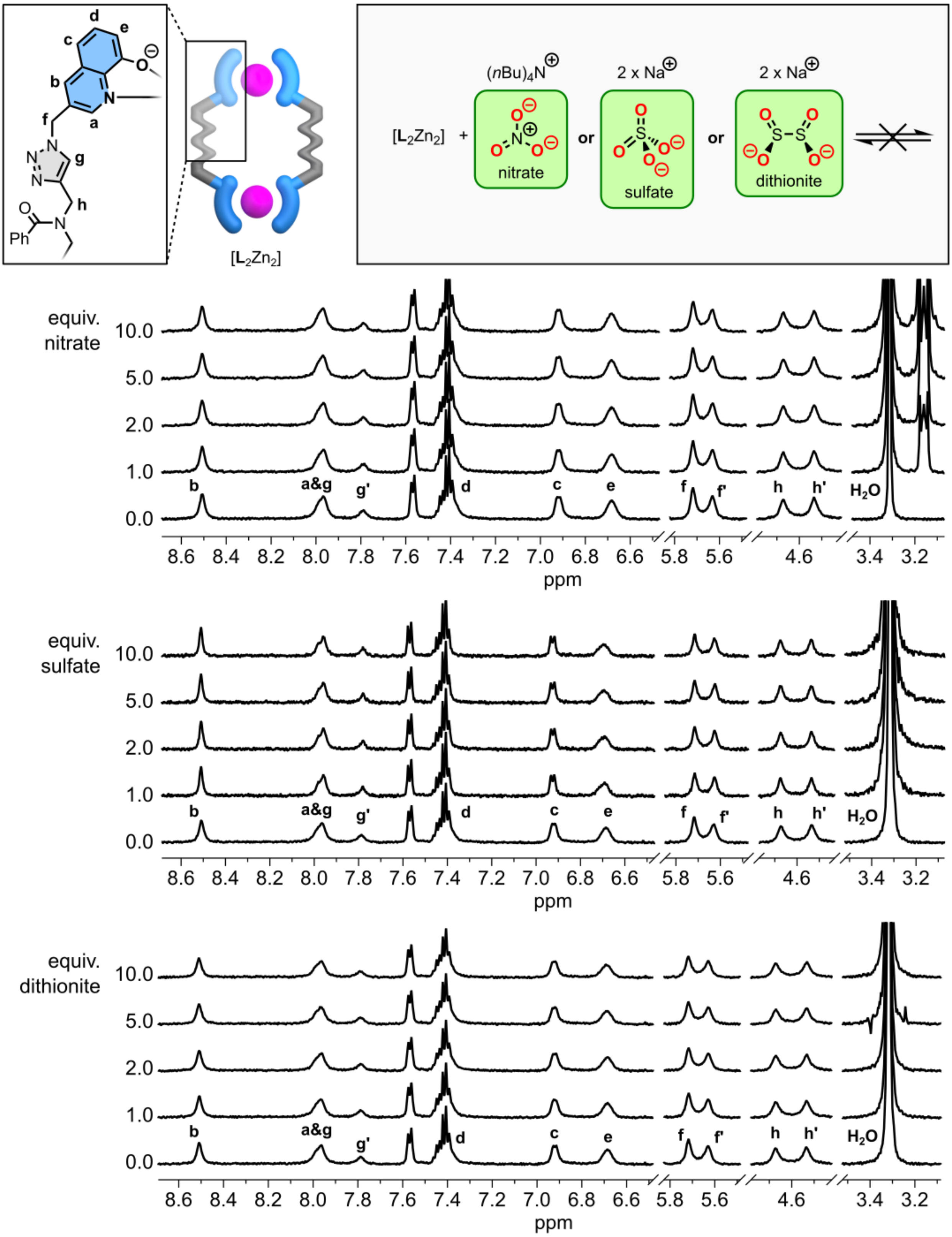

Figure S31: ^{1}H NMR titration (500 MHz, 500 μM, DMSO-d_6, 25 °C) of [$\mathbf{L}_2$Zn$_2$] with tetrabutylammonium nitrate, sodium sulfate and sodium dithionite.

4. Computational studies

4.1. DFT calculations

The following relevant information was used in the input file's header for geometry optimization at the ωB97X-D4[9]/def2-SVP[10,11] level of theory in DMSO (CPCM[12] solvent model applied) with ORCA 5.0.3[13,14]:

! wB97X-D4 def2-SVP Opt SlowConv CPCM(DMSO)

%scf autoTRAH false end

%geom trust -0.1 end

XYZ data for the optimized structure of $[(C2)@L_2Zn_2]^{2-}$ (148 atoms):

Zn -0.93367875677014 -0.58884770834741 2.39795959006068
Zn -0.06926467533533 0.62419665728443 -2.94789640248994
N -4.52067877950102 -3.73008890077641 -3.50707552074735
C -3.31798389779962 -4.28340071235272 -3.77495993786396
C -3.49799612730424 -5.62861413700259 -3.50518717502345
N -4.78661222823198 -5.80211216825522 -3.11197331725856
N -5.39210093935631 -4.65589643469184 -3.11469070600186
H -2.46162073319020 -3.70736848470541 -4.11536244351257
C -2.49397132616693 -6.74913154794813 -3.48502987162263
N -1.58862726438806 -6.67665104074622 -2.33013925232568
C -2.08876925128409 -7.21040816019347 -1.06485430637347
C -3.19775091501647 -6.38662425809139 -0.47404505162618
N -4.36467319371413 -6.93531136152493 -0.04774085017641
N -5.18092731008885 -5.98806427774355 0.29837294259576
N -4.56539090662876 -4.82573049697387 0.10203882944245
C -3.31315710743704 -5.01606143840465 -0.37045933740940
H -2.63592222426217 -4.20526311941864 -0.62492520888366
H -1.91350962034339 -6.75598610715602 -4.41298142848800
H -3.03530657874238 -7.70344062187541 -3.42834895445985
H -2.47303166362516 -8.22594984487071 -1.23902428283927
H -1.23862351522437 -7.27598174641636 -0.37346882393036
H -5.05206390711395 -0.93960334004970 -5.89938454791848
C -4.19195389430248 -0.67656067096353 -5.27493712434560
N -2.03519530519126 0.01339250941656 -3.67613930726644
C -3.96557797527162 -1.34575586546023 -4.09329683968309
C -3.35188867938956 0.39878348575177 -5.67719898123670
C -2.28973268454727 0.73432434429129 -4.80259956673827
C -2.82519362398606 -0.97388360277483 -3.32493474265028
C -3.56231172579797 1.15920812541033 -6.85877217030538

C -2.72937621778813 2.23403571587371 -7.10709824874111
C -1.69157650691337 2.60454274525236 -6.22518167476875
C -1.43501749159473 1.89124503325220 -5.04233020108132
H -1.06622580357468 3.47528246680972 -6.44464951673436
H -4.37732646986262 0.89325053517625 -7.53672624621493
H -2.88161541715874 2.83155045874358 -8.01264500838603
O -0.53124768273885 2.20903762817837 -4.18137535720043
H -2.57568968369662 -1.50606691364277 -2.39900223023329
C -4.97718675355251 -2.33756677472181 -3.57677854849500
H -5.86927921532921 -2.32260210377753 -4.21672288943439
H -5.28875429668059 -2.04681794573864 -2.56278199112074
C -0.56648608497969 -5.76757420518890 -2.25906977798874
O -0.13444062548883 -5.35752137770502 -1.18668326953503
C 0.04285382201172 -5.29898402476413 -3.55124811401517
C 1.24983680244517 -4.36841478045253 -5.89285529461487
C 0.25155599116914 -3.92758738613271 -3.73513323344282
C 0.47235645586548 -6.20696190536171 -4.52742075801221
C 1.08352519826497 -5.74179131112606 -5.69256054048437
C 0.83241230721375 -3.46020989391899 -4.91610554304503
H -0.04393897327654 -3.21892517012178 -2.95479950681239
H 0.33531582472376 -7.28112870920824 -4.37162799557041
H 1.42656719264085 -6.45465512203522 -6.44806033286389
H 0.94502991588175 -2.38011319517458 -5.05320413407721
H 1.71303654624980 -4.00352344380824 -6.81484381293666
H -6.40953779561769 -2.97653687635630 2.72592580631228
C -5.43690945822603 -2.49763895803003 2.57081141551916
N -2.96862908801822 -1.29323692475654 2.17961091354218
C -4.73842189307452 -2.70870983115492 1.40394851253666
C -4.91626141798023 -1.64205875429601 3.58241890266455
C -3.65084992805393 -1.05742130089168 3.33137906759340
C -3.48166056703157 -2.06269123809089 1.25110780345751
C -5.59658667494971 -1.34860571042920 4.79552573241706
C -4.99861653931311 -0.48713417323866 5.69571656470541
C -3.73952243371219 0.10656296292474 5.45534667281731
C -3.01618100872720 -0.15386605148077 4.28075610379829
H -3.30099295240911 0.78241443350841 6.19601403817566
H -6.57295571744910 -1.79893877440009 4.99232459318002
H -5.51557712871085 -0.25092079706744 6.63221933459017
O -1.86790530977329 0.36167805849456 3.99773772677239
H -2.89009526525607 -2.18822344942323 0.33862941824836
C -5.29094313144698 -3.57191482413796 0.29578862479690
H -6.32831758755653 -3.85727046517375 0.50742889055152

H -5.27342711749114 -3.02806194192028 -0.65830488878342
C -0.68251631825129 0.76418706924993 -0.13304328717381
C -0.32029268764360 -0.72928100271923 -0.41699671693469
O -0.98298532371240 1.05556322945429 1.04833637438270
O -0.01927125881791 -1.02061464449044 -1.59823040676662
O -0.62584921444128 1.55436192870161 -1.10130381512491
O -0.37754864582235 -1.51953544180416 0.55118010722422
N 3.56350675201979 4.86234840536794 -0.64975219847575
C 2.31121317185744 5.05409492984201 -0.17807096077325
C 2.19780835162494 6.42473502752452 -0.07324702343849
N 3.36547367764294 6.97212462491069 -0.49920075359840
N 4.18071973927615 6.02394989046345 -0.84517887539743
H 1.63259734208133 4.24411030012083 0.07535837618212
C 1.08969074907056 7.24948919308567 0.51783059545165
N 0.58819757310111 6.71481345031636 1.78219541707440
C 1.49282956099181 6.78514379988421 2.93775475738630
C 2.49622205808843 5.66403627967495 2.95723995892337
N 3.78529139796376 5.83745387262913 2.56546046817541
N 4.39028894727866 4.69097137092850 2.56732609977253
N 3.51817046254612 3.76513813753266 2.95807725913780
C 2.31554448065607 4.31867507583369 3.22575431033204
H 1.45869911113864 3.74261771056175 3.56486304376901
H 0.24002336178650 7.31704468624373 -0.17396536152409
H 1.47525756032396 8.26428218928484 0.69345999630463
H 2.03479936533633 7.73918996161840 2.88268366919910
H 0.91168093131114 6.79118559838556 3.86529847784876
H 5.40634097480985 3.01427005539017 -3.27367545508959
C 4.43390520265713 2.53494312498283 -3.11868565663779
N 1.96604694576111 1.32945068728304 -2.72772647775311
C 3.73565531053956 2.74485016833085 -1.95148168095806
C 3.91324040914483 1.67996390436120 -4.13079307627370
C 2.64809621257365 1.09470681743284 -3.87983627504628
C 2.47913851935650 2.09831614893780 -1.79879486759208
C 4.59327091728685 1.38778030065189 -5.34438043138709
C 3.99523375184634 0.52705269991340 -6.24521235493986
C 2.73645179323474 -0.06735849389745 -6.00488779171646
C 2.01353193913805 0.19170022646033 -4.82977827243772
H 2.29776460943134 -0.74247803795826 -6.74612646042036
H 5.56933657043331 1.83876765489968 -5.54118572165942
H 4.51204219513420 0.29168898158884 -7.18201073152806
O 0.86548332300867 -0.32453326298306 -4.54683664837855
H 1.88780018821996 2.22315456583550 -0.88602807410948

C 4.28785780813109 3.60753925957228 -0.84276354602803
H 5.32584585504631 3.89170208337183 -1.05298023237591
H 4.26830632684831 3.06382611258728 0.11136713074906
C -0.43451359101835 5.80651777599450 1.70926104408458
O -0.86680320579932 5.39894963088528 0.63603764247203
C -1.04548119938129 5.33729685372655 3.00043087728880
C -2.25427870513656 4.40571916982114 5.34067870293969
C -1.25391788707293 3.96578743126551 3.18374768743240
C -1.47578653539316 6.24487584820454 3.97660856505844
C -2.08784710879208 5.77919230846125 5.14108075210532
C -1.83564842837869 3.49789344608187 4.36409912600263
H -0.95704804957329 3.25738155885302 2.40370989310947
H -1.33785025083110 7.31906310385291 3.82169012513271
H -2.43196078611992 6.49175096884422 5.89638053671408
H -1.94778852214029 2.41769015980090 4.50091648438515
H -2.71810971460513 4.04042987684873 6.26219441642064
H 4.04850828173065 0.97522237906827 5.34968046122017
C 3.18844308778426 0.71203938172242 4.72523211179342
N 1.03166259266578 0.02187264300300 3.12649493874829
C 2.96225429216649 1.38082262347403 3.54332532535173
C 2.34807260252940 -0.36291901934668 5.12787190129975
C 1.28596609627102 -0.69860000485486 4.25329681303611
C 1.82189771847516 1.00885210613240 2.77495886367758
C 2.55803813239885 -1.12280900888736 6.30987797667509
C 1.72497033339156 -2.19748459025095 6.55840436618104
C 0.68736091555407 -2.56827508766427 5.67637541915891
C 0.43108729779701 -1.85533057254182 4.49326443727757
H 0.06192952959273 -3.43891896154592 5.89599968248713
H 3.37287705579734 -0.85662400878842 6.98795336331831
H 1.87686425271880 -2.79458357618981 7.46428244376339
O -0.47253777549419 -2.17324230677809 3.63220657922956
H 1.57291952778093 1.54049464837625 1.84855623606059
C 3.97393438075058 2.37231964561978 3.02639471901246
H 4.86664519753088 2.35655712463409 3.66544608467956
H 4.28429045776948 2.08189103291721 2.01193231750125

4.2. MD simulation

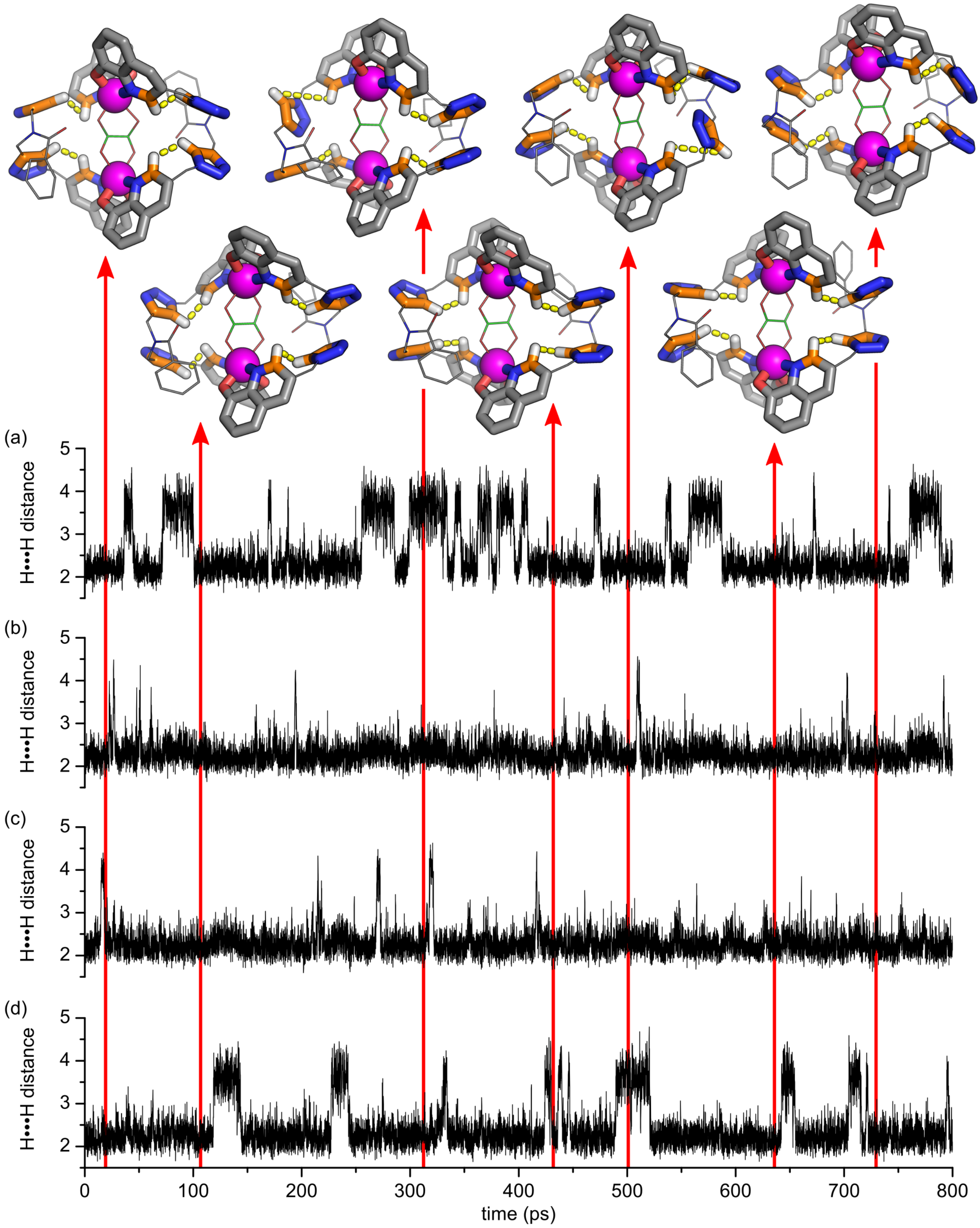

Figure S32: Distance between the triazole proton and the proton in the 2-position of the quinolate unit for all four contacts marked with yellow dashes (a: up left, b: up right, c: down left, and d: down right). Seven snapshots elucidate the possible triazole rotations which take place at $25\,^{\circ}\mathrm{C}$ (xTB 6.6.0[15,16], see relevant information regarding the set-up on the next page).

The following command line was used to start the MD simulation with a prior geometry optimization (below with "...." as placeholder for the file names):

```
xtb "input-file".xyz --chrg -2 --cosmo dmso --omd --input "control-file".ctrl --parallel 20 > "output-file".out
```

The control file contained the following information about the simulation time ($800\,\mathrm{ps}$) and the applied constraints for the quinolate-zinc coordination bonds (oxalate-zinc coordination bonds were not constrained):

```
$md
    time=800
$end

$constrain
    atoms: 1, 56
    atoms: 1, 68
    atoms: 1, 132
    atoms: 1, 144
    atoms: 2, 24
    atoms: 2, 36
    atoms: 2, 100
    atoms: 2, 112
$end
```

5. References

[1] Van Craen, D.; Kalarikkal, M. G.; Holstein, J. J. *J. Am. Chem. Soc.*, **2022**, *144*, 18135-18143.

[2] Hashmi, A. S. K.; Molinari, L.; Rominger, F.; Oeser, T. *Eur. J. Org. Chem.* **2011**, 2256-2264.

[3] Smedley, C. J.; Barrow, A. S.; Spiteri, C.; Giel, M.-C.; Sharma, P.; Moses, J. E. *Chem. Eur. J.* **2017**, *23*, 9990-9995.

[4] Dai, H.; Liu, G.; Zhang, X.; Yan, H.; Lu, C. *Organometallics* **2016**, *35*, 1488-1496.

[5] http://supramolecular.org (Accessed for data fitting in July, August and October, 2023)

[6] Hibbert, D. B.; Thordarson, P. *Chem. Commun.* **2016**, *52*, 12792-12805.

[7] Thordarson, P. *Chem. Soc. Rev.* **2011**, *40*, 1305-1323.

[8] Gans, P.; Sabatini, A.; Vacca, A. *Talanta* **1996**, *43*, 1739-1753.

[9] Najibi, A.; Goerigk, L. *J. Comput. Chem.* **2020**, *41*, 2562-2572.

[10] Weigend, F.; Ahlrichs, R. *Phys. Chem. Chem. Phys.* **2005**, *7*, 3297-3305.

[11] Weigend, F. *Phys. Chem. Chem. Phys.* **2006**, *8*, 1057-1065.

[12] Barone, V.; Cossi, M. *J. Phys. Chem. A* **1998**, *102*, 1995-2001.

[13] Neese, F. *WIREs Comput. Mol. Sci.* **2012**, *2*, 73-78.

[14] Neese, F. *WIREs Comput. Mol. Sci.* **2022**, *12*, e1606.

[15] Bannwarth, C.; Caldeweyher, E.; Ehlert, S.; Hansen, A.; Pracht, P.; Seibert, J.; Spicher, S.; Grimme, S. *WIREs Comput. Mol. Sci.* **2021**, *11*, e1493.

[16] Bannwarth, C.; Ehlert, S.; Grimme, S. *J. Chem. Theory Comput.* **2019**, *15*, 1652-1671.
